# The impact of environmentally friendly supramolecular coordination polymers as carbon steel corrosion inhibitors in HCl solution: synthesis and characterization

**DOI:** 10.1038/s41598-024-51576-9

**Published:** 2024-01-29

**Authors:** M. Eissa, S. H. Etaiw, E. E. El-Waseef, A. El-Hossiany, A. S. Fouda

**Affiliations:** 1 College of Science, Chemistry Department, Al Imam Mohammad Ibn Saud Islamic University (IMSIU), Riyadh, 11623 Kingdom of Saudi Arabia; 2 Department of Chemistry, Faculty of Science, Tanta University, Tanta, 31527 Egypt; 3 Delta for Fertilizers and Chemical Industries, Talkha, Egypt; 4 Department of Chemistry, Faculty of Science, Mansoura University, Mansoura, 35516 Egypt

**Keywords:** Chemistry, Materials science

## Abstract

Two 3D-supramolecular coordination polymers (SCP1 & SCP2) have been synthesized and characterized by physicochemical and spectroscopic methods. In a solution of 1.0 M HCl, SCPs were used to prevent corrosion of carbon steel (CS). The inhibition productivity (%**η**) rises as the synthetic inhibitor dose rises, and the opposite is true as the temperature rises. The study was carried out using chemical (mass loss, ML) and electrochemical ( potentiodynamic polarization, PDP and electrochemical impedance microscopy, EIS) techniques, which showed %η reached to 93.1% and 92.5% for SCP1 & SCP2, respectively at 21 × 10^−6^ M, 25 °C. For the polarization results, SCPs behave as mixed-type inhibitors. With increasing doses of SCPs, the charge transfer resistance grew and the double layer's capacitance lowered. The creation of a monolayer on the surface of CS was demonstrated by the finding that the adsorption of SCPs on its surface followed the Henry adsorption isotherm. The parameters of thermodynamics were computed and explained. The physical adsorption of SCPs on the surface of CS is shown by the lowering values of free energy (∆G^o^_ads_ < − 20 kJ mol^−1^) and increasing the activation energy (E^*^_a_) values in presence of SCP1 & SCP2 than in their absence. Atomic force microscope (AFM) and scanning electron microscopy (SEM) demonstrated the development of a protective thin film of SCPs precipitated on the surface of CS. There is a strong matching between results obtained from experimental and theoretical studies. Results from each approach that was used were consistent.

## Introduction

Due to the many advantages, steel has including: low cost, excellent mechanical properties, durability, strength and versatility, making it an ideal material for use in building and construction, chemical processing, instrumentation manufacturing, marine applications, nuclear fuel power plants, mining, pipelines, oil refining and many other uses. But it suffers from a very serious problem that threatens the global economy, which is the problem of corrosion, especially in an acidic environment^1,2^. In the presence of aggressive media such as HCl, the surface of the CS alloy is damaged and the metal begins to corrode. A 1 molar solution of HCl is considered to be a strong acid, it can be dangerous if not handled properly and may corrode metal but is not toxic. In addition, HCl is one of the acids widely utilized for mechanical purposes, such as corrosive cleaning, corrosive pickling, and corrosive descaling to increase the efficiency of wells in oil, gas production^3,4^. This acid provides CS very corrosive environments. Consequently, the investigation of corrosion-resistant computer systems (CS) is a subject matter of significant theoretical and practical significance. Furthermore, the surface of CS has to be shielded from this unwanted process since, like other metallic materials, it is prone to corrosion. Recently, metal parts in industrial and household appliances have been subjected to less or no corrosion thanks to the use of corrosion inhibitors. A 1 molar solution of HCl is considered to be a strong acid, it can be dangerous if not handled properly and may corrode metal but is not toxic. According to the definition of a corrosion inhibitor, a material is one that, when diluted with corrosive solutions can reduce or even stop the interaction of metals with the solutions. Inhibitors are organic substances that contain heteroatoms such as nitrogen, oxygen and sulfur atoms as well as many pre-existing bonds that increase their adsorption ability on the metal surface and prevent corrosion^5–7^. The synthetic organic inhibitors are not only readily available, inexpensive, and made from renewable resources, but are also inexpensive^8,9^. Many researchers have studied the inhibition of CS dissolution in acidic environments using various types of synthetic composites^10^, the organic composites have been used to prevent the corrosion of mild steel^11^, aluminum^12^, copper^13^ stainless steel and steel^14^. The improved corrosion resistance of several metals has also been found in inorganic compounds^15^. MOFs have been developed for various applications including catalysts^16^, sensors^17^, drug delivery^18^, batteries^19^, separation^20,21^, and corrosion ^22,23^. There are tiny studies in the literature showing the use of MOFs as an effective corrosion inhibitor”, most of these studies in this field have used Schiff base monomers or polymer complexes as acidic corrosion inhibitors^24–28^. Three ligands were introduced to generate five Cd (II) base complexes and the corrosion-inhibiting properties of these complexes on mild steel in 15% HCl were determined^29^. A copper (I) coordination polymer containing the corrosion inhibitor 1H–-benzotriazole: poly [3-benzotriazole-3N1:N2:N3-copper (I)] was used as the corrosion inhibitor^30^. Abiola^31^ reported that 3-(4-amino-2-methyl-5-pyrimidylmethyl)-4-methylthiazolium chloride at a concentration of 0.5 mol/L and 5 mol effectively prevented hydrogen evolution and corrosion of structural steel in 1 mol./L HCl at 30 °C. The results reported by Di et al.^32^ used BTA-Cu-MOF coating showed that the MOF effectively diffuses into the epoxy resin and acts as an anti-corrosion substance for metals. To protect mild steel in saline solutions, Ali et al.^33^ prepared an anti-corrosion coating from samarium (III) nitrate and [bis (phosphonomethyl) amino] methylphosphonic acid. An even coating of this thin layer forms on the surface of the steel”. For the first time, Berdimurodov et al.^34^ utilized 6-aminopenicillanic acid sodium gossypol (APASG) as anti-corrosion properties for St2 steel in a 1 M HCl + 1 M KCl solution^34^. The obtained findings demonstrated that, at moderate temperatures (303–333 K), APASG is a good anti-corrosion inhibitor (97% at 100 mg/L/0.104 mM). Zhu et al.^35^ investigated the corrosion prevention efficacy of the synthesized N-doped carbon quantum dots NCQDs on Q235 steel in a 1 M HCl solution. On Q235 steel, the synthesized NCQDs had a strong anticorrosion impact, with 150 mg/L of NCQDs exhibiting a corrosion inhibition efficacy of 95.4%. N-hydroxypyrazine-2-carboxamide (NHP) was employed by Dewangan et al.^36^ as a novel, green, and efficient corrosion inhibitor to prevent mild steel surface corrosion in an aggressive 1 M HCl solution. According to the acquired data, NHP's inhibitory efficacy was 93.51% at 200 ppm at 298 K. In order to investigate the inhibitory impact of two bis-phosphonic acids on XC48 steel corrosion against 1 M HCl, Ouksel et al.^37^ used a one-pot reaction of 2,6-bis (hydroxymethyl)-4-methoxyphenol or 4-chloro-2,6-bis (hydroxymethyl) phenolphosphite. In 0.5 M H2SO4, an aromatic hydrazone derivative known as (E)-1-(3-nitrobenzylidene)-2-(p-tolyl) hydrazine (E-NBPTH) was studied^38^ as a corrosion inhibitor for XC48 carbon steel. Using modified techniques, Chafai et al.^39^ examined the inhibitory effect of a novel α-aminophosphonic derivative on the corrosion of XC48 carbon steel in 0.5 M H_2_SO_4_.

Due to SCPs have significant advantages such as: (i) uniform structure, ultra-high porosity, customizable composition and easy to functionalize surface, thanks to the wide applications of SCPs in many areas such as anti-corrosion coatings, and (ii) the use of an organic–inorganic hybrid and (iii) SCP1 and SCP2 have supramolecular dimensions compared to most heterocyclic compounds. To date, inhibitors for [metal–organic frameworks (SCPs)], particularly for the protection of carbon steel, have been less reported. This encourage us to investigate the use of SCP1 and SCP2 as corrosion inhibitors for CS in 1 M HCl solutions using ML and electrochemical techniques (PDP, EIS), a SEM and AFM techniques were used to characterize the surface shape of the CS with and without the addition of inhibitors.

## Experimental

### Components and the solution

The chemical structure of CS used in this study is “C 0.28, Mn 0.6, P 0.04, Si 0.003 and iron rest (weight %). Analytical HCl (37%) was diluted with double distilled water to obtain 1.0 mol/L HCl for use under severe conditions”. The inhibitors used were prepared from stock solution of 1 × 10^−3^ M which was prepared from solution of (1:10) dimethyl formamide: ethanol. The dosage of SCPs used in our study was ranged from 1 to 21 × 10^−6^ M by dilution by distilled water.

### Inhibitors and chemicals

#### Synthesis of _∞_^3^[Cu_2_(CN)_4_(Ph_3_Sn)_2_. dmqox], SCP1^40,41^

A solution of 90 mg (0.31 mmol) “K_3_[Cu(CN)4] in 5 ml H_2_O was added dropwise with gentle stirring to a solution of 366 mg (0.95 mmol) Ph_3_SnCl and 50 mg (0.31 mmol) given and dmqox in 10 mL of warm acetonitrile. A yellow precipitate formed immediately. After filtration, washing with small amounts of H_2_O and acetonitrile, and drying overnight, about 314 mg (93.4% expressed as K_3_ [Cu (CN)_4_]) of a yellow precipitate was obtained. All previous attempts to grow individual SCP1 crystals suitable for X-ray examinations have so far resulted in the formation of a microcrystalline powder. Elemental Analysis: Calculated” Calc. for C_50_H_40_N_6_Cu_2_Sn_2_ (%): C, 55.24; H, 3.68; N, 7.73; Cu, 11.69, Found: C, 55.33; H, 3.62, N, 7.67; Cu, 11.56.

#### Synthesis of_∞_^3^ [Cu (CN)_2_ Me_3_Sn·qaz], quinazoline (qaz), SCP2^40,41^

An aqueous solution of 90 mg (0.31 mmol) “K_3_[Cu(CN)_4_] was added with gentle stirring to a solution mixture containing 189 mg (0.95 mmol) Me_3_SnCl and 40 mg (0.31 mmol) quinazoline ( qaz) contained in 10 mL Acetonitrile. After two weeks, the yellow prismatic crystals began to separate from the initially clear solution. After filtration, washing with a small amount of cold water and acetonitrile, and drying overnight, 84 mg of SCP2 was recovered. Elemental analysis data for C_13_H_15_N_4_CuSn is analytical data. Calculate. C, 38.14; H.3.67; N, 13.69; With 15.54 and Section C 38.20; H., 3.74; N, 13, 6; Z, 15:44”.

### Methods

#### Chemical tests ((ML measurements)

Corrosion rate and braking efficiency are calculated according to the static mass loss measurement standard ASTM G1-03 (reapproved in 2017)^42^. Seven samples are cut and polished with different types of sandpaper (100, 600, 1500, and 2000). “The samples were cleaned, dried and washed with a small amount of acetone to remove grease and other organic contaminants and then placed in solutions prepared with various doses of synthetic compounds (SCP1 and SCP2). Previously prepared part samples were fully immersed in a 250 mL beaker containing various concentrations of inhibitor/acid solution. Each measurement was carried out for 3 h at a temperature between 20 and 45 °C.The samples are left in the solutions for 30 min. They were then collected, dried and weighed before being incorporated back into the solutions. The steps are repeated until the end of the experiment, which lasts 3 h. To achieve the best repeatability, the experiments were performed three times. ML is repeatedly measured and plotted. The temperature range is between 25 and 45 °C. ML was calculated by knowing the weight of the samples before and after immersion and the corrosion rate (υ) is obtained by Eq. (1)

1 $$ \upsilon = \left( {{\text{m}}_{{\text{o}}} {-}{\text{m}}} \right)/{\text{st}} $$

where the weights of the samples before and after dipping are *m*_*0*_ and *m*, respectively, s is the area (cm2) and t is the time of immersion (min). The following formula can be used to determine S, the surface area of the coins with the corresponding inhibitory efficiency from the ML approach (*η*_w_):

2 $$ \% \upeta_{{\text{w}}} = \uptheta \times {1}00 \, = \, (\upsilon_{{\text{o}}} {-}\upsilon )/\upsilon_{{\text{o}}} \times {1}00 $$

where ϴ surface coverage is, and *ν*_*o*_ and are the corrosion rates of coin specimens in HCl electrolyte solutions without and with a specific additive concentration of SCP1 & SCP2 (g m^−2^ min^−1^).

#### Electrochemical measurements

The working electrode that was previously examined is used in the approach, which is a cell with three electrodes^12^. “This electrode has a 1 cm^2^ exposed surface area and is composed of CS metal, the process of manufacture for which was previously disclosed^12^. The electrode is prepared in line with the test prior to use, followed by ML and the use of a saturated calomel electrode (SCE) as the reference. Next, the Pt foil auxiliary electrode is used. After 30 min of immersion in the OCP solution, the CS electrode reaches the stability criterion. PDP curve measurements were done in the 250 mV potential range using an OCP scanning rate of 0.2 mVs^−1^. The current density is utilized for the measurements in this test. Electrochemical impedance spectroscopy (EIS) measurements were carried out in the frequency range of 10^5^ Hz to 0.01 Hz using an AC excitation pulse with amplitude of 10 mV. Electrochemical frequency modulation (EFM) was investigated by sending a signal with a 10 mV capacity across two sinus waves with a frequency range of 2 to 5 Hz^43^. This equipment is quick and non-destructive. Corrosion current density (i_corr_), causal factors CF-2 and CF-3, and Tafel slopes (ß_a_ and ß_c_) were found through the higher peaks^44^.

The Gamry Potentiostat/Galvanostat/ZRA (PCI4-G750) was the instrument used in the electrochemical research. Gamry includes a computer for data collection together with the DC105 DC corrosion software, EIS300 EIS software, EFM140 for EFM software. Echem Analyst version 5.5 was used to plot, compute, and synthesize the data”. At 25^0^C, all electrochemical experiments were conducted. When doing electrochemical measurements, the following formulas are used to determine the inhibitory efficiency:

3 $$ \left( {\eta_{{\text{p}}} } \right) = \uptheta \times {1}00 = \left( {{1} - i{{{\text{corr}}({\text{inh}})}} } \right)/ i {{{\text{corr}}(0)}} \times {1}00 $$

4 $$ \left( {\eta_{{\text{R}}} } \right) = \uptheta \times {1}00 = \left( {{ 1} - R_{{{\text{ct}}(0)}} } \right)/R_{{c{\text{t}}({\text{inh}})}} \times {1}00 $$

Here i_corr(0)_ and i_corr(inh)_ are the corrosion current densities of coins in aggressive media, respectively before and after the addition of the inhibitor. *R*_*c*t(inh)_ and *R*_*c*t(0)_ are the charge transfer resistances in the presence and absence of the inhibitor, respectively.

### Surface examinations

“The CS samples used for surface morphological tests were prepared in 1 M HCl acid (blank) and 21 × 10^−6^ M inhibitors at room temperature for one day after physical abrasion with various sanding sheets up to 1,200 grit, then after 10 minutes After immersion, samples were gently washed with distilled water, dried thoroughly, and placed into prepared samples that were evaluated using a scanning electron microscope (SEM) and an atomic force microscope (AFM)”.

### Computational methods

#### Quantum chemical calculations

The calculations from quantum chemical theories were used as a helpful tool for comprehending the material characteristics and the corrosion inhibition procedure, based on a variety of methodologies^45,46^. Density Functions Theory (DFT) was applied using the Dmol3 module in Material Studio software (version 7.0) to explore the whole geometrical optimization of the inhibitors under investigation. Both the efficiency and the corrosion inhibition behavior of the two derivatives are expressed by their molecular and electronic structures. Quantum characteristics such as electronegativity (χ), dipole moment, chemical potential (μ), smoothing (σ), absolute hardness (η), and the energies of the highest occupied (HOMO) and lowest unoccupied (LUMO) molecular orbitals were estimated using the DFT approach.

#### Monte-Carlo simulations

The best configuration of the investigated compounds on the surface of Fe (1 1 0) was assessed using Monte Carlo simulations. The Fe (1 1 0) crystal surface is thought to be the most stable surface utilized in this simulation, according the literature^47^. In order to examine the adsorption of uncharged and protonated inhibitor molecules, 100 water molecules were used to mimic the solvent action during the corrosion process. First, geometrical optimization of the water and inhibitor molecules was carried out using the quote module. The substrate-adsorbate system's configuration space was searched using a Monte-Carlo method to find low-energy adsorption locations where the temperature is progressively lowered. The total energy, adsorption energy, and substrate-adsorbate energy were calculated using the Monte Carlo simulation.

## Results and discussion

### (ML) measurements

**Figure 1:**
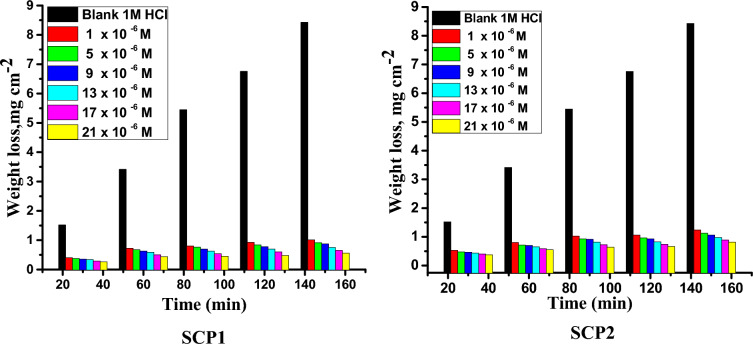
ML-time diagrams for the dissolution of the CS in 1.0 M HCl solution in the absence an attendance of altered doses of SCPs at 25 °C.

For a specified dipping period, the ML of the samples was measured. This method involves dipping the sample in an acid solution for a certain period of time, after which the sample's level of corrosion is determined^48^. The SCP1 and SCP2 were put through various temperatures and dosages in 1.0 molar hydrochloric acid during the experiments. The results of an investigation that lasted 120 min are shown in Table 1. The results showed that the corrosion rate (C.R.) reduced with increasing inhibitor doses while the rate of inhibition increased with doses from 1 × 10^−6^ to 21 × 10^−6^ M (Fig. 1). Inspection of Table 1 shows that by increasing the temperature the C.R. will significantly accelerate and thus %η will fall. The % improvement with higher synthetic inhibitor dose can be used to qualify the adsorption of the inhibitor layer on the CS surface. The oxygen and nitrogen atoms of the inhibitor molecules as well as the electrons in the aromatic rings that create free electron pairs make up this layer. This shows that the inhibitor molecules of SCP1 and SCP2 are adsorbed on the metal surface and may mainly belong to physical adsorption, that is, low temperature is beneficial for the adsorption of inhibitors inhibition on the CS surface. The competing adsorption of acid ions on the metal surface becomes more intense at higher temperatures, which improves metal corrosion and lowers inhibitive effectiveness. The percentage inhibition **% η**) was in this order: SCP1 is superior to SCP2. Equation (2) was used to calculate the percentage inhibition of the CS and the degree of surface coverage (ϴ). Table 1 summarizes the information that C.R. and **% η** collected from synthetic compounds (SCP1&SCP2) at various temperatures and dosages.

**Table 1:** Outcome data of ML of the CS in 1.0 M HCl solution for various doses of synthetic inhibitors (SCPs) after 120 min and at altered temperatures.

Temp. °C	Conc., × 10^6^ M	SCP1		SCP2	
		C.R. × 10^−2^ mg cm^−2^ min^−1^	% η	C.R. × 10^−2^ mg cm^−2^ min^−1^	% η
25	1	0.77 ± 0.0017	86.3	0.89 ± 0.0016	84.3
	5	0.70 ± 0.0014	87.6	0.80 ± 0.0017	85.8
	9	0.65 ± 0.0022	88.5	0.77 ± 0.0019	86.3
	13	0.58 ± 0.0021	89.6	0.69 ± 0.0020	87.8
	17	0.50 ± 0.0021	91.1	0.61 ± 0.0023	89.1
	21	0.40 ± 0.0024	93.0	0.50 ± 0.0024	91.1
35	1	1.38 ± 0.0020	77.0	1.10 ± 0.0016	81.6
	5	1.21 ± 0.0017	79.8	0.98 ± 0.0020	83.7
	9	1.05 ± 0.0023	82.4	0.89 ± 0.0014	85.2
	13	0.92 ± 0.0018	84.7	0.85 ± 0.0013	85.7
	17	0.74 ± 0.0019	87.7	0.78 ± 0.0012	87.0
	21	0.59 ± 0.0016	90.1	0.71 ± 0.0014	88.2
45	1	3.14 ± 0.0111	74.3	2.33 ± 0.0019	80.8
	5	2.92 ± 0.0021	76.1	2.08 ± 0.0021	82.9
	9	2.43 ± 0.0012	80.1	1.90 ± 0.0012	84.4
	13	1.96 ± 0.0014	83.9	1.83 ± 0.0023	85.0
	17	1.57 ± 0.0017	87.1	1.66 ± 0.0024	86.4
	21	1.28 ± 0.0016	89.5	1.50 ± 0.0017	87.7

#### Adsorption isotherm behavior

**Figure 2:**
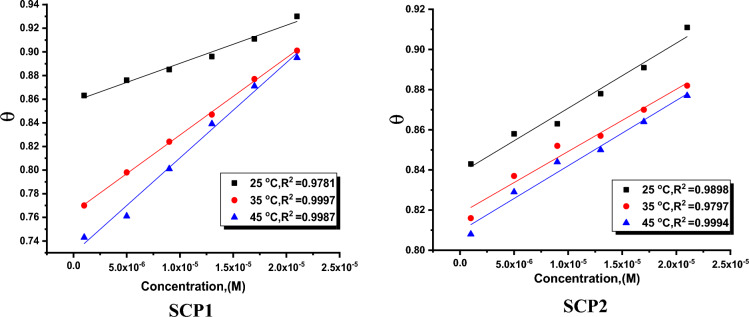
Plotted as (θ) versus C of the SCP1 & SCP2 for dissolution of CS in 1.0 M HCl solution from ML tests at 25 °C.

The enhanced **% η** with a higher doses of synthetic inhibitors can be used to be qualified the adsorption of an inhibitor layer on the CS surface. This layer is composed of pairs of free electrons that are formed by the oxygen and nitrogen atoms in the inhibitor molecules and the π-electrons of the aromatic rings. Physical, chemical, and reterodonation (where the inhibitor molecules' unoccupied molecular orbitals and the surface metal atoms’d-orbitals are involved) adsorption are the three main types of adsorption that can be occurred^49^. The adsorption process becomes more intense if the inhibitor molecules contain heteroatoms like nitrogen, sulphur, or oxygen because these atoms emit one pair of electrons that lead to electrostatic adsorption on the surface of the CS and the formation of an insoluble layer that slows down the dissolution of CS. Using a suitable isothermal technique, the adsorption conditions of the synthesized compounds on the CS surface may be established. To ascertain whether adsorption isotherms were most suitable, data for ML tests were created in several mathematical adsorption isotherm expressions using data recorded at various temperatures. According to this study's findings, Henry isotherm is the most suitable adsorption isotherm for the adsorption of SCPs on CS surface (Fig. 2). This isotherm demonstrates how the θ and inhibitor dosage are related”.

5 $$ {{\theta}} = {\text{ KC}}$$

This equation C and K_ads_ use symbols to describe, respectively, the dosage of the synthesized chemicals and the adsorption process' equilibrium constant. “The Henry isotherm's R^2^ value was precisely closer to the unit, according to the investigating data in Table 2. The Henry adsorption isotherm thus provided a good description of the inhibitor adsorption behavior seen on the surface of CS. In Table 2, the term "K_ads_" stands for "adsorption strength" and "displays the molecular strength of the adsorbed layer." Low values of K_ads_ reflect weaker interactions between the inhibitor molecules that are adsorbing to the CS surface, however this only indicates that the solvent molecules can easily remove the inhibitor molecules from the metal surface”. In the current work, the adsorption of molecules in synthetic compounds (SCP1&SCP2) was investigated using free energy and the following formula:

6 $$\Delta{\text{G}}_{{{\text{ads}}}}^{0} = - {\text{RT ln }}\left( {{55}.{5} \times {\text{K}}_{{{\text{ads}}}} } \right)$$

**Table 2:** lists the Henry parameters for the adsorption of synthetic composites on CS surface at various temperature conditions.

Compound	Temp., °C	Adsorption parameter			
		K_ads_, Mol/L	− ∆G°_ads_ kJ/ mol	− ∆H°_ads_ kJ/ mol	− ∆S°_ads_ J/mol K
SCP1	25	3.2 ± 0.0212	12.8 ± 0.0202	39 ± 0.2125	43 ± 0.2021
	35	6.5 ± 0.0187	15.1 ± 0.0167		49 ± 0.1723
	45	8.6 ± 0.0198	16.3 ± 0.0147		51 ± 0.1625
SCP2	25	2.9 ± 0.0142	12.6 ± 0.0187	42 ± 0.1758	42 ± 0.1658
	35	3.1 ± 0.0122	13.2 ± 0.0241		42 ± 0.1457
	45	3.3 ± 0.0172	13.7 ± 0.0198		43 ± 0.1111

The amount of water in the bulk solution is represented by the number 55.5 in this equation when expressed in mol/L^50^. “Table 2 shows the adsorption properties of SCPs. The data in the Table showed the spontaneous adsorption and stability of the double layer adsorbed on the metal surface, which are verified by the negative values of the free energy obtained. The results from ∆G^0^_ads_ show that the sort of adsorption that leads to physical adsorption is known to occur when ∆G^0^_ads_ values are less than 20 kJ / mol, which is consistent with the data found^51^. This is supported by the literature review. Using the Vant't Hoff equation, the following equation for ∆H°_ads_ and ∆S°_ads_ can be measured”:

7 $$ {\text{Ln}}\;{\text{K}}_{{{\text{ads}}}} = \frac{{ - \Delta {\text{H}}_{{{\text{ads}}}}^{^\circ } }}{{{\text{RT}}}} + const $$

**Figure 3:**
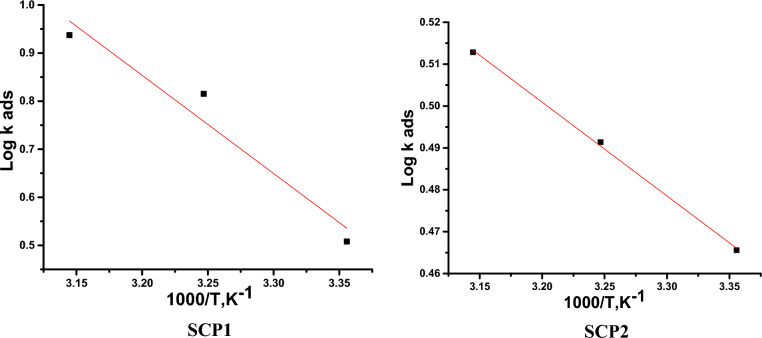
Log K_ads_ versus 1/T for the liquefaction of CS in 1.0 M HCl in the existence of SCP1 &SCP2.

The correlation between log K_ads_ and 1/T is shown in Fig. 3. The standard adsorption enthalpy change was calculated from the following balance (− ∆H^o^_ads_), and the entropy value was given (− ∆S^o^_ads_):

8 $$ \Delta {\text{G}}^{{\text{o}}}_{{{\text{ads}}}} = \, \Delta {\text{H}}^{{\text{o}}}_{{{\text{ads}}}} - {\text{ T }}\Delta {\text{S}}^{{\text{o}}}_{{{\text{ads}}}} $$

We are aware that the enthalpy values in the current investigation are negative and therefore the SCPs inhibitor adsorption molecules are exothermic. The exothermic process can refer to either physical or chemical adsorption, even though the H^o^_ads_ values are less than 100 kJ/mol, indicating that the adsorption is mostly physical”^52^.

#### Effect of temperature

The effect of temperature on the dissolution of CS samples employed in the synthetic compounds and dipped in 1.0 M of hydrochloric acid was studied in the current experiment both with and without various doses of synthetic compounds (1 × 10^−6^–21 × 10^−6^ M) at 25–45 °C. "It has been demonstrated that the C.R. rises as the temperature rises. From the slope of the graphs, the activation energy (E^*^_a_) was calculated using the Arrhenius Eq. (9)^53^.

9 $$ {\text{Log C}}.{\text{R}}. \, = {\text{ log A }}{-}{\text{ E}}_{{\text{a}}}^{*} /{2}.{3}0{\text{3RT}} $$

**Figure 4:**
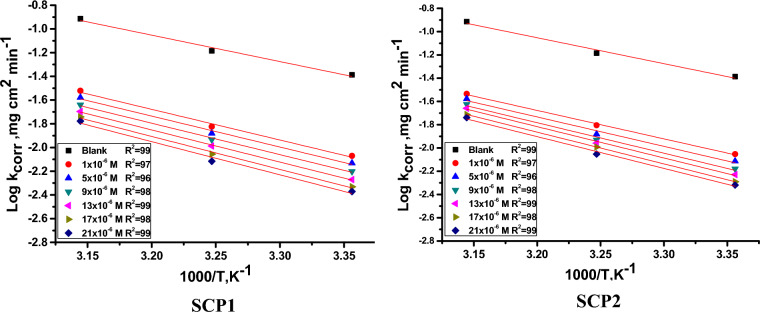
shows plots of the dissolution of CS with and without different dosages of SCPs (log k_corr_ vs. 1/T).

In contrast, the Arrhenius exponential factor was represented by the letter (A). Arrhenius plots [log (C.R.) against 1/T] are displayed in Fig. 4. The Arrhenius equation, which has a slope of [- E^*^a /2.303R], is demonstrated by the plot, which yields straight lines with almost unit regression coefficients. The transitional state equation was used to calculate the variations in entropy and enthalpy^54^.

10 $$ {\text{Log }}\left( {{\text{C}}.{\text{R}}./{\text{T}}} \right) \, = \, [{\text{log}}\left( {{\text{R}}/{\text{Nh}}} \right) \, + \Delta{\text{S}}_{{\text{a}}}^{*} /{2}.{3}0{\text{3R}}] - \Delta{\text{H}}_{{\text{a}}}^{*} /{2}.{3}0{\text{3RT}} $$

**Figure 5:**
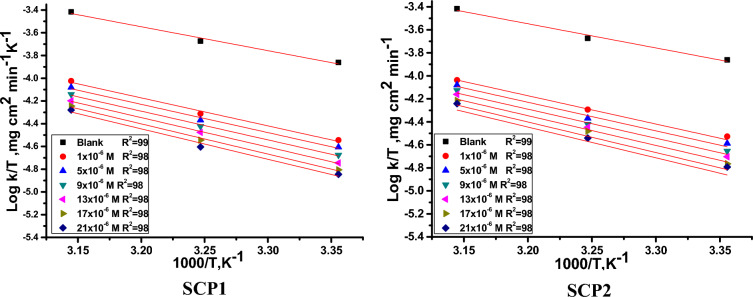
illustrates the plots of (log k_corr_/T) versus 1/T for the dissolution of CS with and without modified dosages of SCPs.

By plotting log (C.R/T) versus 1000/T, Fig. 5 shows the SCPs compounds in their transitional state. Slopes determine enthalpy, and the intersection of the lines [log(R/Nh) + ∆S^*^_a_ /2.303R] determines the activation entropy of the process. The findings analysis was compiled in Table 3. Data in Table 3 showed that the E^*^_a_ increased as the SCP's chemical dose was increased and was found to be higher than that of the control solution. The physical adsorption of the SCPs compounds on the CS surface is what causes this increase. The results in Table 3 showed that SCPs compounds result in negative entropy values, and these negative values showed that the activated complex in the rate-determining state preferred association over dissociation^55^. The results confirm the existing thermodynamic relationship between E^*^_a_ and ∆H^*^_a_, which characterizes a unimolecular process”^56^.

11 $$  {\text{E}}_{{\text{a}}}^{*}  - \Delta {\text{H}}_{{\text{a}}}^{*}  = {\text{ RT}}     $$

**Table 3:** lists the parameters of thermodynamics obtained from the Arrhenius and transition state equations.

Inhibitor	Conc., × 10^6^ mol/L	Activation parameters		
		Ea*	ΔH*	− ΔS*
		kJ/mol	kJ/mol	J/mol/k
Blank	0.0	42.6 ± 0.2012	40.2 ± 0.2124	137 ± 0.2245
SCP1	1	49.5 ± 0.1704	47.1 ± 0.2245	127 ± 0.1758
	5	49.9 ± 0.1657	47.5 ± 0.1754	126 ± 0.1452
	9	50.7 ± 0.1453	48.3 ± 0.1457	125 ± 0.1245
	13	51.8 ± 0.1856	49.5 ± 0.1124	122 ± 0.1758
	17	53.2 ± 0.1553	50.8 ± 0.1457	119 ± 0.1741
	21	53.5 ± 0.1245	51.2 ± 0.1234	119 ± 0.1475
SCP2	1	46.7 ± 0.1145	44.3 ± 0.1024	135 ± 0.1485
	5	48.3 ± 0.1345	45.9 ± 0.1754	131 ± 0.1756
	9	50.1 ± 0.1457	47.7 ± 0.1242	127 ± 0.1754
	13	51.5 ± 0.1574	49.1 ± 0.1714	123 ± 0.2123
	17	52.2 ± 0.1547	49.8 ± 0.2127	121 ± 0.2134
	21	52.8 ± 0.1573	49.9 ± 0.1654	122 ± 0.1789

The computed value (2.4 at 25 °C) and expected value (in Table 3) are very similar. The inhibitors thereafter had a similar effect on E^*^_a_ and ΔH^*^_a_.

### Electrochemical measurement

#### Open circuit potential (OCP) measurements

**Figure 6:**
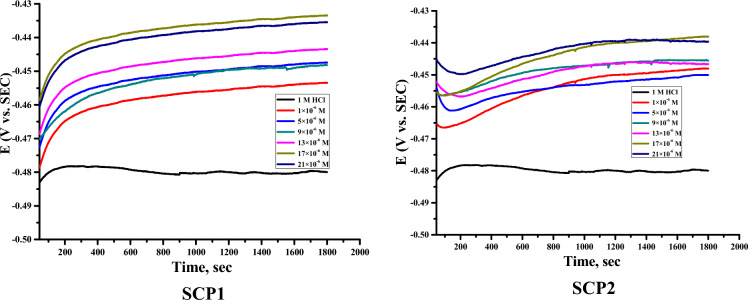
Variation of E_OCP_ versus time for CS in the 1.0 M HCl solutions in the absence and presence of various concentrations of the SCP1 & SCP2 at 25 °C.

The OCP versus time curves for C-steel corrosion in 1.0 M HCl are shown in “Fig. 6 in both the absence and presence of various concentrations of derivatives of supramolecular coordination polymers (SCP1 and SCP2). The potential for an unconstrained solution is seen to decline over time and stabilize at a value of − 435 mV/ SCE for SCP1 and − 430 mV/ SCE for SCP2, respectively, at about 200 s. This behavior can be explained by the corrosive compounds that emerge on the surface of the CS as a result of its degradation. In other words, when supramolecular coordination polymers (SCP1 & SCP2) are present at concentrations between 1 and 21 × 10^−6^ M, the potential of SCP2 decreases, then increases, and then swiftly stabilizes over time, whereas the potential of SCP1 increases at first before reaching steady state after around 200 s. The disintegration of the oxide coating and the development of a protective film on the metallic surface can both be used to explain these phenomena. In inhibited solutions, the potentials obtained moved to values more positive than those found in uninhibited solutions, according to a careful inspection of the OCP curves”. This suggests that these substances have cathodic inhibitory effects.

#### PDP tests

**Figure 7:**
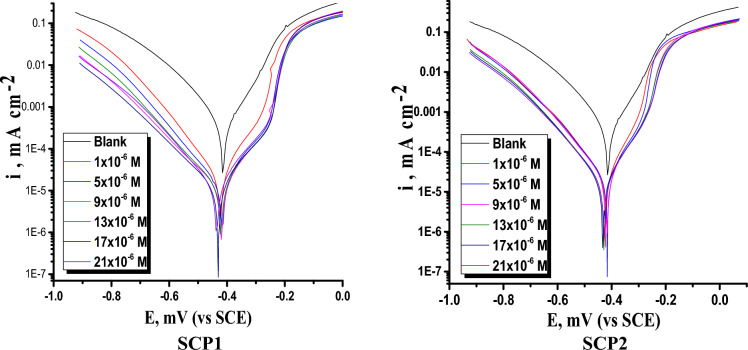
Shows the PDP bends for CS dissolution at 25°C in the absence and adding of altered dosages of SCP1 and SCP2.

In 1.0 M HCl medium at 25 °C, the PDP test is conducted with and without varying concentrations of synthetic substances (SCP1 & SCP2). “Table 4 displays the PDP parameters data, and Fig. 7 shows drowning. Figure 7 shows the polarization curves with and without SCP1 and SCP2 present. The cathodic and anodic overpotentials were decreased, and the major parallel displacement was moved to more negative and positive values with the addition of SCP1 and SCP2, respectively. The activation-controlled nature of hydrogen change is demonstrated by the parallel cathodic and anodic Tafel curves, and the presence of SCP1 & SCP2 has no effect on the reduction and dissolution processes^57^. Corrosion parameters were derived from the cathodic and anodic potential versus current density characteristics of the Tafel potential area^58,59^. The data in Table 4 showed that the current density decreases with increasing doses of the SCP1 and SCP2 compounds, indicating that a layer has formed on the CS surface. Additionally, the cathode and anode Tafel slopes do not indicate a significant change despite the presence of different dosages of SCP1 and SCP2 chemicals (Fig. 7). This can be explained by the fact that corrosion is caused by blocking the CS's surface's active spots. If there is a difference in corrosion potential (E_corr_) values between the presence and absence of synthetic molecules of more than 85 mV, the inhibitor molecules are classified as cathodic or anodic, but this did not happen since the displacement in corrosion potential values is less than 85 mV. However, a little change in the E_corr_ (63 mV) and a small change in (ß_a_ & ß_c_) on the rise in the dose of the used inhibitors revealed that these compounds work as mixed type inhibitors^60,61^. The corrosion current density dropped when the doses of synthetic compounds were raised. The (η_p_ %) was calculated from PDP curves using Eq. 3. Molecular size, shape, and contact mode are just a few of the factors that influence inhibitory efficiency^62^. The relationship of molecule size, structure, and functional group explains the high level of inhibitory activity observed. SCP1 came first in the ranking of (η_p_ %), followed by SCP2 (SCP1 > SCP2).

**Table 4:** **P**arameters determined from the PDP for the corrosion of CS at 25 °C in both the absence of adding altered dosages of SCP1 and SCP2.

Comp	Conc. × 10^6^ M	i_corr_, μA cm^−2^	− E_corr_, mV vs SCE	β_a_ mV dec^−1^	− β_c_ mV dec^−1^	C.R. mpy	Ɵ	% η
SCP1	1	108 ± 0.1758	423 ± 0.1571	116 ± 0.2132	180 ± 0.2214	28	0.781	78.1
	5	97 ± 0.1578	421 ± 0.1174	123 ± 0.1755	191 ± 0.1452	20	0.804	80.4
	9	71 ± 0.1647	428 ± 0.1147	122 ± 0.1458	186 ± 0.1758	13	0.856	85.6
	13	57 ± 0.1879	430 ± 0.1624	127 ± 0.1245	179 ± 0.1124	8	0.885	88.5
	17	50 ± 0.1147	431 ± 0.1479	118 ± 0.1475	182 ± 0.1457	7	0.899	89.9
	21	34 ± 0.1456	436 ± 0.1247	124 ± 0.1234	179 ± 0.1785	6	0.931	93.1
SCP2	1	115 ± 0.1457	423 ± 0.1124	125 ± 0.1475	190 ± 0.1467	47	0.767	76.7
	5	102 ± 0.1658	431 ± 0.1657	121 ± 0.1587	181 ± 0.1234	35	0.794	79.4
	9	78 ± 0.1754	421 ± 0.1874	119 ± 0.1957	183 ± 0.1231	31	0.842	84.2
	13	62 ± 0.1457	423 ± 0.2153	120 ± 0.2123	189 ± 0.1245	28	0.874	87.4
	17	55 ± 0.1147	418 ± 0.2034	121 ± 0.1475	181 ± 0.1897	23	0.889	88.9
	21	40 ± 0.1437	420 ± 0.1485	119 ± 0.1957	192 ± 0.1425	20	0.919	91.9

#### EIS tests

**Figure 8:**
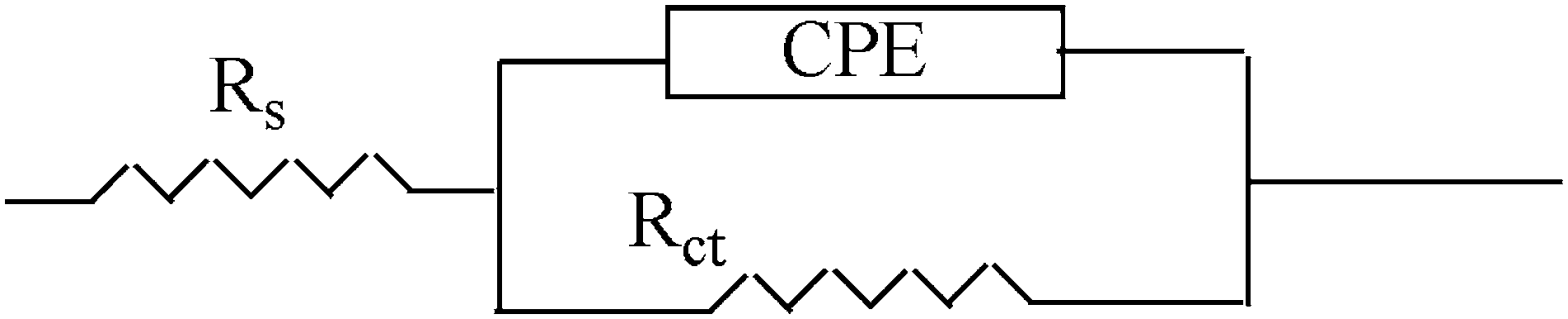
The impedance data is fitted using the equivalent circuit.

**Figure 9:**
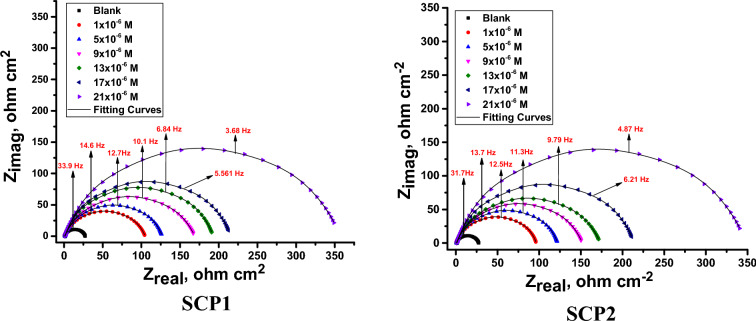
The Nyquist bends for CS dissolution 1.0 M HCl with and absence of altered doses of SCP1 &SCP2 at 25 °C.

**Figure 10:**
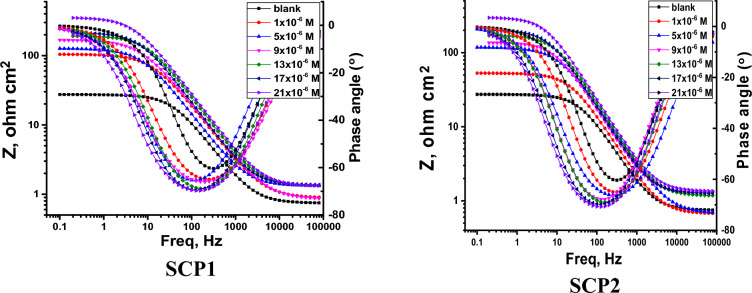
The Bode graphs for the CS dissolving in 1.0 M HCl, both with and absence of modified dosages of SCP1 and SCP2.

At 25 °C in an acid medium, EIS studies with and without SCP1 & SCP2 inhibitors were carried out. Figure 8 displays the impedance values of the CS as discovered via comparable circuit studies using various SCP1 and SCP2 chemical dosages. Constant phase elements (CPE) are used in this circuit to produce several homogenizations that are not the best for preventing electrode corrosion, such as grain boundaries, surface impurities, and roughness (a decrease in polishing). Using the equation below, the capacitance double layer (C_dl_) was produced”:

12 $$ {\text{C}}_{{{\text{dl}}}} = \, \left( {{1}/{2}\pi {\text{f}}_{{{\text{max}}}} {\text{R}}_{{{\text{ct}}}} } \right) $$

where f_max_ is the maximum frequency. Table 5 displays the results from the impedance data for CS in 0.1 M hydrochloric acid in both the presence and absence of various dosages of the SCP1 and SCP2 compounds. The graphs that Nyquist and Bode discovered for CS when SCP1 and SCP2 were not present and when they were present at various doses are displayed in Figs. 9, 10, respectively. According to the Nyquist plots in Fig. 9, the circle's radius increases when the dose of SCP1 and SCP2 compounds rises. Consequently, an increase in the thickness of the electrical double layer (C_dl_) causes the increase in the charge transfer resistance (R_ct_) in corrosion processes. As a result of the single time constant in impedance spectroscopy, this illustrates a single capacitive loop in the applied frequency range. Charge transfer resistance was correlated with a higher frequency range, but film resistance produced by an inhibitor layer was correlated with a lower frequency range^63,64^. In our case, during the adsorption process while the inhibitor was present, a very thin layer formed on the metallic surface. Because of frequency dispersion and microscopic roughness of the electrode surface, Nyquist curves for CS in an uninhibited solution flatten in the presence of inhibitors. The semi-circular Nyquist plot of an unconstrained solution indicates that the charge transfer resistance appears to have been the controlling component in the CS corrosion process. However, to illustrate the polarization resistance (R_p_) between the metal and outer Helmholtz plane in the presence of inhibitors, the depressed semi-circular Nyquist plot is taken into consideration. According to the current finding, a high resistance has been established as a result of the SCP1 and SCP2 compounds adhering to the CS-solution/interface^65^. The heterogeneity of the CS surface and the frequency dispersion are responsible for the Nyquist curves' departure from semicircles^66^. Table 5 shows that the data for C_dl_ decreases as the dose of SCP1 and SCP2 compounds increases. This decrease in C_dl_ can be attributed to a local dielectric constant decrease or an increase in the thickness of the electrical double layer^67^. “The n value assesses the roughness or inhomogeneity of the solid surface. The n value increases in the 1 M HCl electrolyte relative to the reference electrolyte when SCP1 and SCP2 are combined (0.978–0.990), which can be interpreted as a little reduction in surface heterogeneity^68,69^. This is caused by SCP1 and SCP2 compound molecules adhering to the solution's CS/interface. From Eq. 4, the EIS parameters and (% *η*_R_) were computed. The low Chi-squared values (Table 5) obtained for all of the results show that there is good agreement between the fitted results and the experimental data. Chi-squared was used to measure how accurately the fitting result was achieved. Figure 9 makes it obvious how well the experimental and theoretical curves are fitted”. These results are generally in agreement with the potentiodynamic polarization results.

**Table 5:** lists the EIS parameters for dissolving CS in 1.0 M hydrochloric acid both with and absence of several dosages of SCP1 and SCP2.

Comp	Conc., × 106 mol/L	Yo, µΩ^−1^ s^n^ cm^−2^	n	Rct, Ω cm^2^	Cdl, µFcm^−2^	% ηR	Goodness of fit (χ2)
Blank	0	189	0.976	27 ± 0.1758	165 ± 0.2132	–	17.33 × 10^−3^
SCP1	1	170	0.978	104 ± 0.1452	155 ± 0.1789	74.4	20.12 × 10^−3^
	5	157	0.980	126 ± 0.1245	144 ± 0.1578	78.8	19.54 × 10^−3^
	9	135	0.982	168 ± 0.1475	125 ± 0.1425	84.1	18.24 × 10^−3^
	13	112	0.987	192 ± 0.1238	106 ± 0.1823	86.1	16.24 × 10^−3^
	17	93	0.988	214 ± 0.1421	89 ± 0.2128	87.5	19.55 × 10^−3^
	21	88	0.989	354 ± 0.1238	85 ± 0.1245	92.5	24.28 × 10^−3^
SCP2	1	163	0.983	95 ± 0.2012	150 ± 0.1124	71.9	21.79 × 10^−3^
	5	156	0.984	115 ± 0.1245	146 ± 0.1758	76.8	19.87 × 10^−3^
	9	148	0.986	150 ± 0.2321	140 ± 0.1852	82.2	18.78 × 10^−3^
	13	142	0.987	172 ± 0.1758	135 ± 0.1254	84.5	14.57 × 10^−3^
	17	116	0.989	202 ± 0.2178	111 ± 0.2128	0.868	16.75 × 10^−3^
	21	103	0.990	325 ± 0.1458	99 ± 0.1987	0.918	21.5410^−3^

#### EFM tests

**Figure 11:**
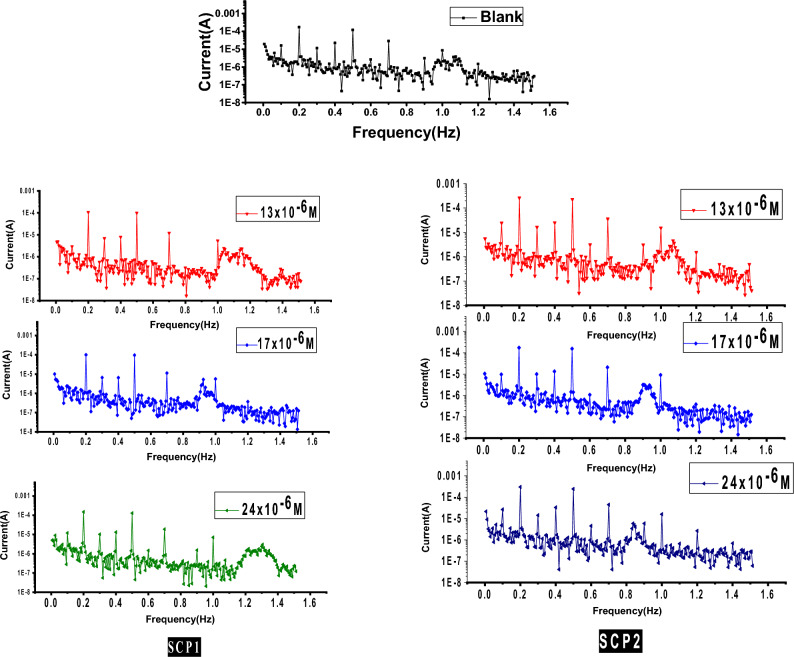
EFM spectra for CS dissolving in 1.0 M HCl at 25 °C with and absence of changed dosages of SCP1.

EFM stands out for its unusual speed and accuracy in calculating current data^70^. Figure 11 displays the EFM of CS in 1.0 M HCl solution together with different dosages of the synthetic inhibitor SCP1. Similar curves were also obtained for a second SCP2 (not shown). The higher current peaks can be used to measure EFM parameters like (CF-2 and CF-3), (c and a), and (i_corr_). The fact that the CF is more similar to the accepted data supports the accuracy of the calculated data^71,72^. The percentage, which was computed and given in Table 6, rises when the synthetic compound is improved. The results in Table 6 show that when concentration rises, there is a discernible fall in current density, which increases percent. This method was used to obtain the following information: There is a discernible decrease in the corrosion current's strength and an obvious rise in the% η with an increased dose of synthetic chemicals. The percentage obtained by applying ML, PDP, and EIS techniques is in the best agreement with that found by EFM tests. The percentage attained with ML, PDP, and EIS techniques is the closest to that found by EFM tests.

**Table 6:** EFM data for CS dissolution 1.0 M HCl with and absence of changed doses of SCP1 &SCP2.

Comp	Conc., × 10^6^ mol/L	i_corr_ µAcm^−2^	β_a_, mVdec^−1^	-βc, mVdec^−1^	C.Rmpy	CF−2	CF−3	θ	%(ηE)
Blank	–	543 ± 0.2111	125 ± 0.1754	153 ± 0.2012	255	1.8	3.1	–	–
SCP1	1	208 ± 0.1745	119 ± 0.1654	147 ± 0.2122	88	2.0	3.0	0.617	61.7
	5	152 ± 0.1457	111 ± 0.1254	143 ± 0.2023	79	1.8	2.9	0.720	72.0
	9	128 ± 0.1241	113 ± 0.1475	137 ± 0.1981	61	2.2	2.8	0.764	76.4
	13	82 ± 0.1457	112 ± 0.12571	151 ± 0.1651	37	1.9	3.0	0.849	84.9
	17	72 ± 0.2021	129 ± 0.2321	155 ± 0.1524	27	2.1	3.1	0.867	86.7
	21	48 ± 0.1451	110 ± 0.1777	148 ± 0.1421	21	2.0	3.2	0.912	91.2
SCP2	1	219 ± 0.13521	113 ± 0.1932	154 ± 0.1654	99	2.0	3.0	0.597	59.7
	5	162 ± 0.1221	110 ± 0.2012	139 ± 0.2211	89	1.9	2.9	0.702	70.2
	9	133 ± 0.1457	118 ± 0.1242	144 ± 0.2012	72	1.8	3.0	0.755	75.5
	13	83 ± 0.2141	112 ± 0.1128	139 ± 0.1427	60	2.0	2.9	0.847	84.7
	17	74 ± 0.1457	111 ± 0.1635	152 ± 0.1654	32	2.1	3.0	0.864	86.4
	21	52 ± 0.1234	120 ± 0.1532	150 ± 0.1247	28	2.0	2.9	0.904	90.4

### Surface analysis

#### Scan electron microscopy (SEM) analysis

**Figure 12:**
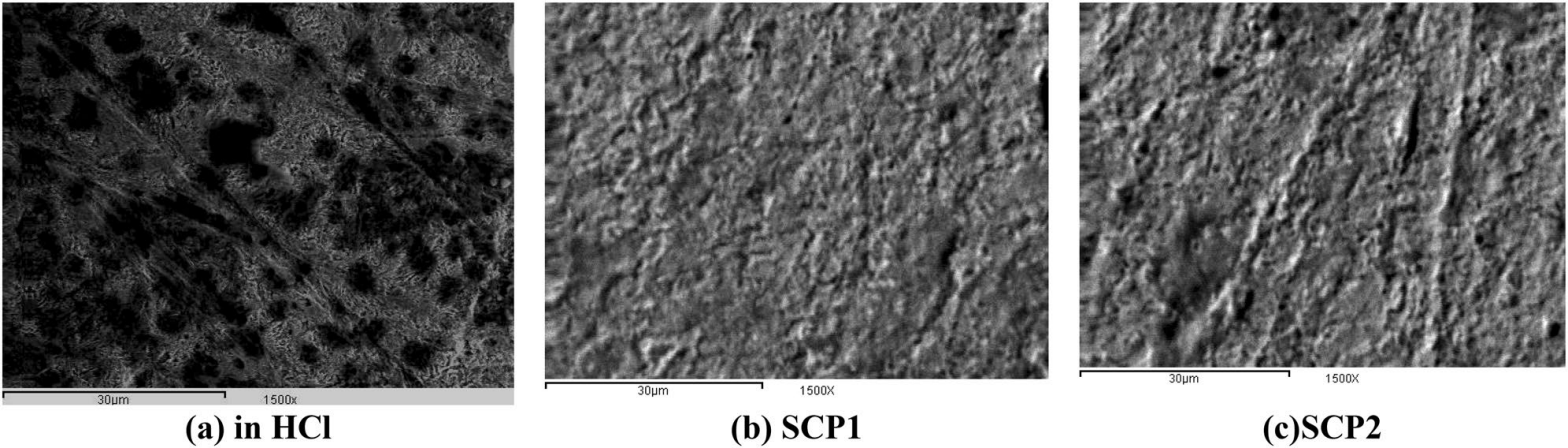
SEM photograph of CS + 1.0 mol/L HCl solution (**a**), and presence (**b**, **c**) of an optimum doses 21 × 10^−6^ M of SCP1 & SCP2, correspondingly.

Using SEM, the morphology of the steel surface was examined to ascertain whether the inhibition was brought on by the development of an organic coating. The surface with a protective film was examined using SEM on the CS specimens after 24 h at 25 °C in 1.0 mol/L HCl solution alone and with adding the optimal concentration (21 × 10^−6^ mol/L) of SCPs (Fig. 12). After being submerged in the corrosive liquid, the surface of the CS metal surface became increasingly corroded with numerous deep cracks (Fig. 12a). This is because the metal in the test solution has dissolved. On the other hand, after the addition of (SCPs) to the test media, the surface became smoother with tiny, deep fractures (Fig. 12b, c). These data suggest that the inhibition is brought on by the development of a persistent, adherent deposit that stops the electrolytes from reaching the steel surface^73^.

#### Atomic force microscope (AFM) analysis

**Figure 13:**
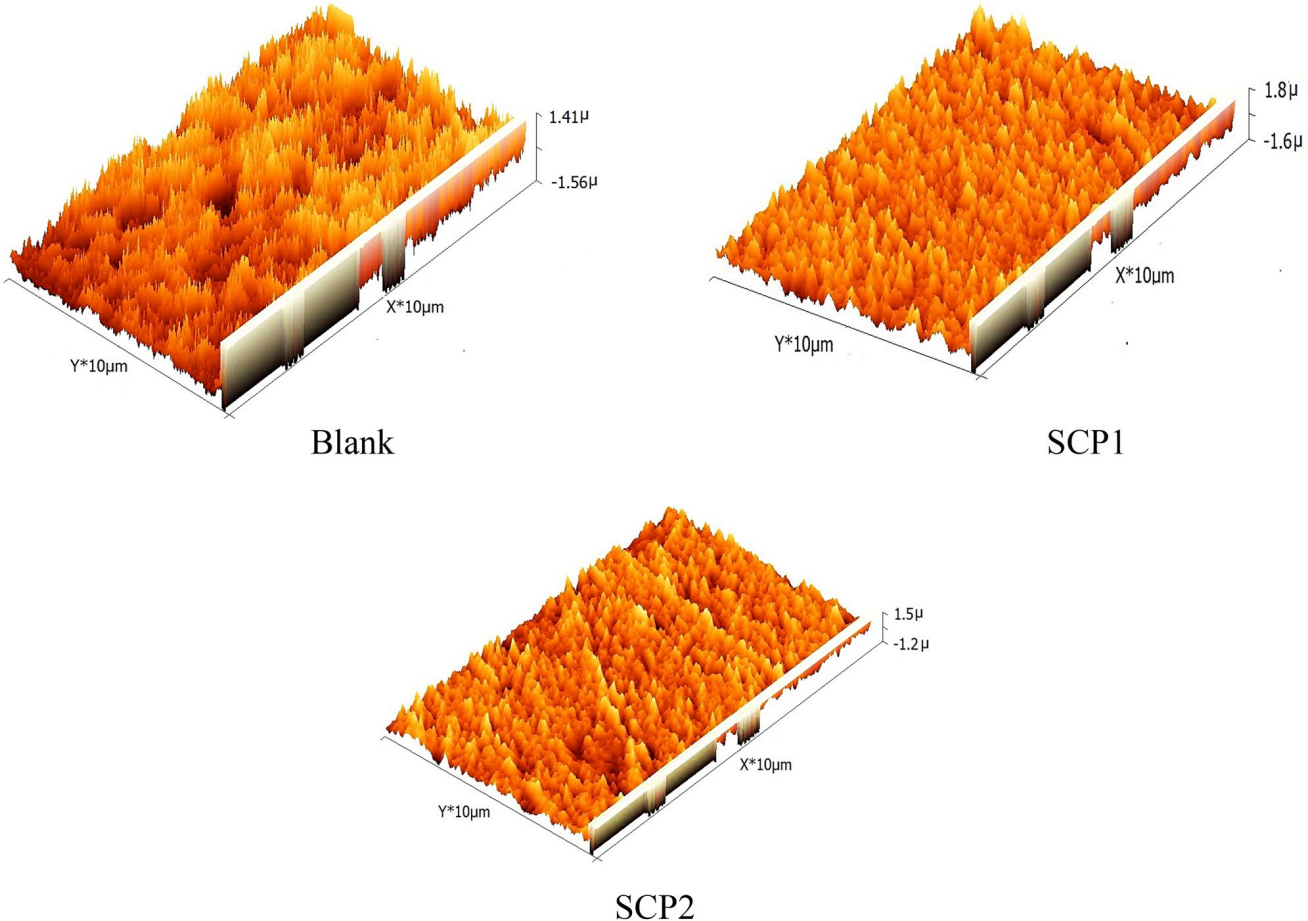
AFM analyses on CS with and absence of 21 × 10^−6^ M SCPs for 1 day's involvement.

One may assess the surface roughness of coupons on an angstrom scale thanks to the atomic or near-atomic resolution surface topography afforded by the AFM technique. “This approach is effective for examining the surface analysis of both inhibited and uninhibited carbon steel surfaces. A 3-D AFM image of the exterior of CS subjected to 1.0 M HCl for 24 h with and without the optimal SCPs level measured is shown in Fig. 1. After being submerged in 1.0 M HCl without an inhibitor for 24 h, the common roughness of the surface of C steel was measured and determined to be 467.57 nm. In Fig. 13, a 3D image of the CS surface is shown while being exposed to the highest doses (21 × 10^−6^ M) of SCP1 and SCP2, bringing the roughness to 91.3 and 102.7 nm, respectively”. This decrease in roughness suggests that the surface of CS has grown a protective film^74^.

### Theoretical analysis

#### Quantum chemical parameters

**Figure 14:**
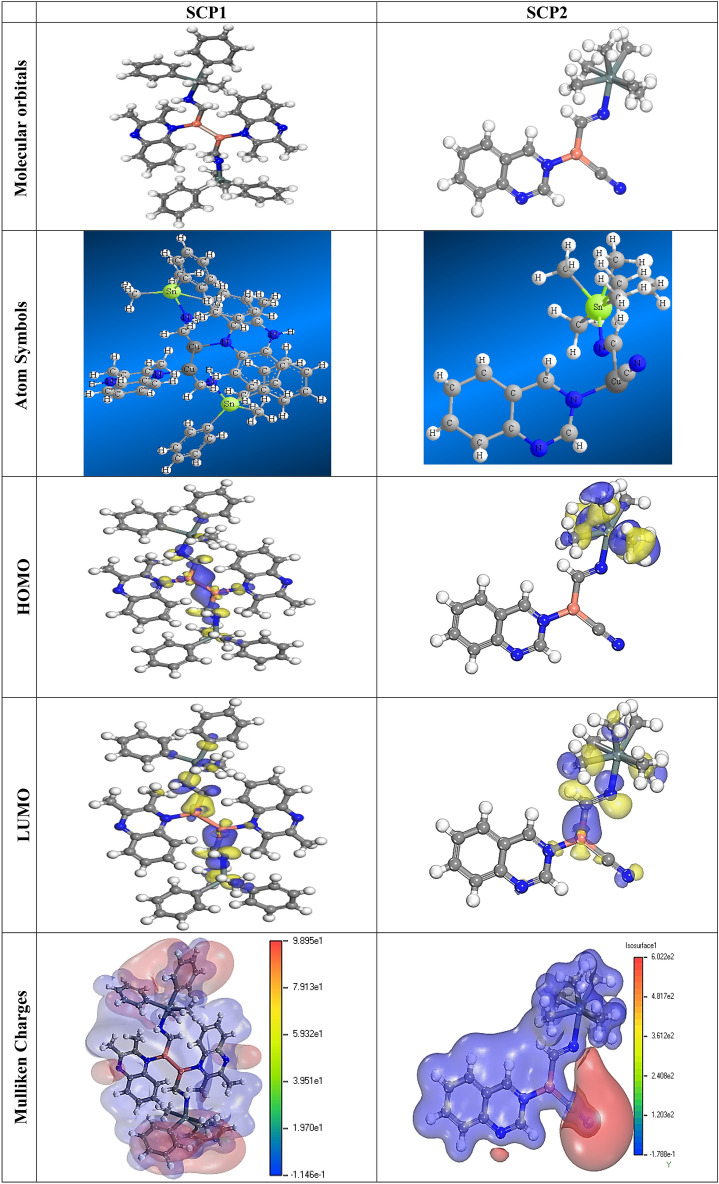
The optimized geometrical structure, (HOMO), and (LUMO) of the tested SCPs at DMol3 using Material Studio software (version 7.0).

The lower energy band gap value, “which is represented in the energy band gap ΔE_g_ (ΔE = E_HOMO_ E_LUMO_), indicates that organic molecules are highly reactive and exhibit excellent corrosion behaviour on the surface of CS. An analysis of the impact of SCPs molecule's orientation on inhibition performance was conducted using density function theory (DFT). As shown in Fig. 14, the optimized geometry, HOMO surface, and LUMO surface of studied inhibitors can be found. The parameters HOMO (E_H_), LUMO (E_L_), and dipole moment (μ) for MOFs gradients were directly obtained from DFT (Table 7). Equations 13–18 were used to calculate the energy gap (ΔE), electronegativity (χ), global hardness (η), global softness (σ), the fraction of electron transfer (ΔN) and back-donation (ΔE back-donation)”, was calculated as Koopmans’s theorem^1^ from the next balance:

13 $$ \mu = - \chi = - \frac{{I_{p} + E_{A} }}{2} $$

14 $$ \chi = \frac{{I_{p} + E_{A} }}{2} $$

15 $$ \eta = \frac{{I_{p} - E_{A} }}{2} $$

16 $$ \sigma = \frac{1}{\eta } $$

17 $$ \omega = \frac{{\mu^{2} }}{2\eta } $$

18 $$ \Delta E_{back\;donation} = - \frac{\eta }{4} $$

**Table 7:** Quantum chemical data for SCPs under study.

Compound	SCP1	SCP2
*-EHOMO, eV*	4.305	4.725
*-ELUMO, eV*	4.202	4.432
*ΔE, eV*	0.103	0.293
*IP, eV*	4.305	4.725
*EA, eV*	4.202	4.432
${\varvec{\chi}}$*, eV*	4.254	4.579
*η , eV*	0.051	0.147
*σ , eV*	19.417	6.826
*ω*	175.653	71.545
*∆N*	26.665	8.265
*ΔEback-donation*	– 0.013	– 0.037
*Dipole moment (Debye)*	2.45	21.7

Numerous articles^75,76^ have discussed how higher values of “E_HOMO_ and lower values of E_LUMO_ determine the greater electron-donating and accepting abilities of an inhibitor. Inhibitors are more reactive when a lesser value of ΔE is present. In this instance, SCP1' ΔE value is lower while higher values for SCP2. In comparison to SCPs molecules, these values suggest that SCP1 molecule has a high degree of reactivity. Metals and inhibitors can be understood using the number/fraction of electron transfer (ΔN). If the ΔN value of an inhibitor is higher, it is found to have a stronger capability of donating electrons to metallic surfaces. Compared to SCPs molecules, SCP1 exhibits greater amounts of ΔN in the gaseous phase, indicating that SCP1 exhibits a stronger inhibitory effect.

#### Monte Carlo (MC) simulation

**Figure 15:**
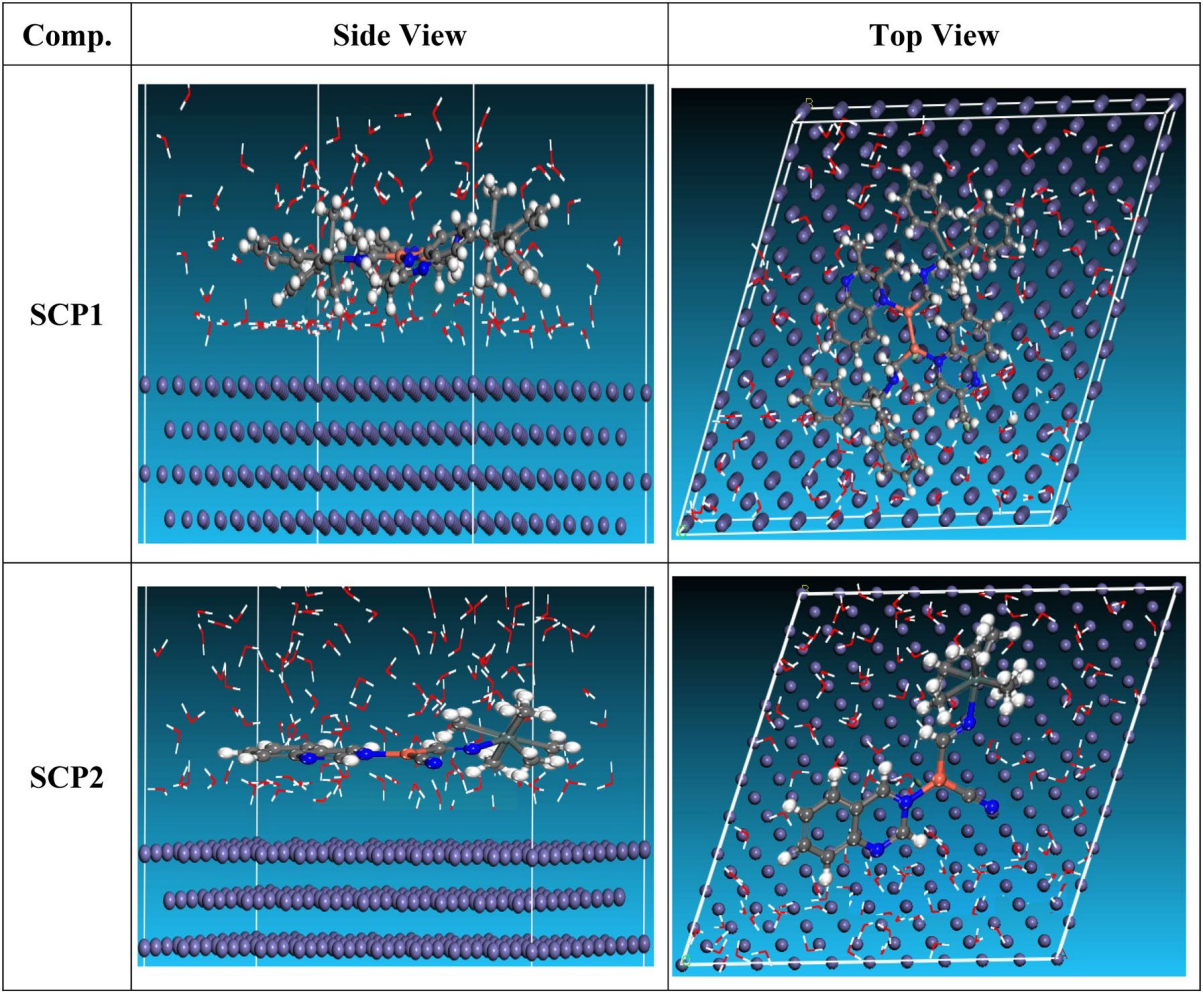
Adsorption shapes of the SCPs molecules on CS surface using Material Studio software (version 7.0).

MC modeling is a good method for calculation the most stable adsorption conformations of a SCPs. Figure 15 illustrates the simulation findings for the investigated SCP, which are described in Table 8. Figure 15 depicts the adsorbed molecule's most favorable confirmation on the CS metal surface (1 1 0). “Furthermore, the molecules stated are adsorbed on the metal surface from the motive, which is rich in inhibitory molecule electrons. The interactions between the occupied orbitals of the examined SCPs and the vacant orbitals of CS (110), which are reflected by energy adsorption values (E_ads_), of the rigid energy (E_rigid_), of the deformation energy (E_def_), and energy ratio values (dE_ads_/dN_i_) of the inhibitors, which is equivalent to the energy of substrate-adsorbate configurations where one of the adsorbate components has been removed are collected in Table 8. Adsorption energy values that are more negative indicate a highly stable and strong connection between adsorbed molecules and metal. When two materials are mixed during the adsorption process, an electron, ion, or molecule (adsorbent) is attached to the solid surface, adsorption energy is defined as declining energy^77,78^. As shown in Table 8, the greater adsorption energy of SCP1 rather than SCP2 on the hardened Fe surface predicts heavy adsorption of SCP molecules, forming a stable adsorbed layer that protects the iron from decomposition. The tabulated adsorption energies are -3868.456 and -3598.362 kcal/mole for SCP1, SCP2 respectively. The outputs show that the two inhibitors are efficient adsorptive inhibitors taking in respect that the better one is SCP1 which is attuned with the experimental results”. Based on theoretical modeling it’s obvious that SCPs based proved to be powerful inhibitors for the CS which is confirmed by experimental and spectral investigation.

**Table 8:** MC parameters of adsorption of SCPs molecules on CS (110) surface.

Structures	Adsorption energy	Rigid adsorption energy	Deformation energy	Compound dE_ad_/dNi	H2O dE_ad_/dNi
CS (1 1 0)/Inhibitor SCP1/H_2_O	– 3868.456	– 3669.886	196.57	– 283.67	– 12.31
CS (1 1 0)/Inhibitor SCP2/H_2_O	– 3598.362	– 3418.992	179.37	– 253.83	– 8.97

### Anti-corrosion mechanism

The initial stage of the inhibitory action in acid media is adsorption on the metal surface^59^. The majority of studies on inhibition postulate that the unoccupied d-orbitals of the metal and the inhibitor's π-electrons will form a donor–acceptor surface complex^79^. SCP2 has two aromatic rings and four N atoms, whereas inhibitor SCP1 contains two aromatic rings and six N atoms. Inhibitors can either be cations (i.e., protonated species) or neutral substances in aqueous acidic solutions. There are two main categories of adsorption that might be considered. The removal of water molecules from the metal surface, the sharing of electrons between N-atoms and Fe, as well as between π-electrons from aromatic rings and the open d-orbitals of Fe, are all steps in the chemisorption process that might lead to the neutral form of the inhibitor attaching to the metal surface. On the other hand, it is well known that the CS surface is positively charged in acidic environments^80^. Protonated inhibitors have a strong time adhering to the positively charged CS surface due to electrostatic repulsion. Since chloride ions are more readily adsorbed, they provide the solution an excess negative charge and favor more protonated inhibitor adsorption due to the lower number of hydrations they possess. In other words, the inhibitors' ability to inhibit may be improved by the synergistic interaction between Cl^−^ and the inhibitors. When the protonated inhibitors are deposited on the metal surface, the following things could occur: The development of a coordinate bond due to the partial transfer of electron from N-atoms to the metal surface and the protonated inhibitors may combine with newly formed Fe^2+^ ions on the CS surface to produce a metal-inhibitor complex [Inh-Fe]^(2+z)+^^81^. These complexes might bind to the surface of the CS utilizing Van der Waals forces, where they will create a corrosion-preventing shielding film”. The film covers both the anodic and cathodic reactive sites on the CS surface, inhibiting both reactions simultaneously. SCP1 > SCP2 in percentage inhibition, this is due to i) the higher molecular weight of SCP1 than SCP2 ii) the presence of 6 donating N atoms in SCP1 than 4N atoms in SCP2 and iii) the presence of double Cu and Sn atoms in SCP1 than in SCP2 and as known the effectiveness of the sacrifice is increased by including additional electropositive metals in the organic framework. On the other hand, the organic structure creates a shield over the metal surface that prevents corrosion (Supplementary file 1).

## Conclusions

The following conclusions may be drawn from the values we gained from theory and experimentation:

1. The synthesis and characterization of the supramolecular coordination polymers (SCPs) were conducted.
2. By increasing the concentration of SCPs the η% increases and vice versa with raising temperature.
3. These substances exhibit mixed sort inhibitory behavior, as seen by the PDP curves.
4. These chemicals adhere to the Henry model and are physically adsorbed on the CS surface.
5. The development of a protective coating from the SCPs on the CS surface was confirmed by SEM and AFM measurements.
6. There is agreement between theoretical investigations, electrochemical measurements, and ML.

### Supplementary Information

Supplementary Information.

## Data Availability

The authors confirm that the data supporting the findings of this study are available within the article and/or its supporting file.
