# Supplementary material for: The impact of environmentally friendly supramolecular coordination polymers as carbon steel corrosion inhibitors in HCl solution: synthesis and characterization
Source: Sci Rep. 2024 Jan 29;14:2413. doi: 10.1038/s41598-024-51576-9 (PMC10825159; doi:10.1038/s41598-024-51576-9)
Supplement: Supplementary file 1 — Supplementary Information. [file 41598_2024_51576_MOESM1_ESM.docx]

**The impact of environmentally friendly supramolecular coordination polymers as carbon steel corrosion inhibitors in HCl solution: synthesis and characterization**

**M. Eissa^1^** [delete]**, S. H. Etaiw**^2^, **E. E. El-Waseef**^2^**, A. El‑Hossiany ^3,4^, A. S. Fouda^4*^**

**^1^**College of Science, Chemistry Department, Al Imam Mohammad Ibn Saud Islamic University (IMSIU), Riyadh 11623, KSA

^2^Department of Chemistry, Faculty of Science, Tanta University, Tanta-31527, Egypt

^3^Delta for Fertilizers and Chemical Industries, Talkha, Egypt

^4^Department of Chemistry, Faculty of Science, Mansoura University, Mansoura-35516, Egypt, email: [asfouda@hotmail.com](mailto:asfouda@hotmail.com); Fax: +2 050 2202264 Tel: +2 050 2365730

**Characterization of Inhibitors (SCP1 and SCP2) ^[1-6]^**

**Characterization and Structure of SCP1**

1) Infrared Spectra of ∞3[ Cu2(CN)4(Ph3Sn)2.dmqox] "SCP1"

The presence of the ternary adducts, Ph_3_Sn^+^ cation, Cu_n_(CN)_m_ building block and dmqox, in the structure of _∞_^3^[ Cu_2_(CN)_4_(Ph_3_Sn)_2_.dmqox] are confirmed by the IR spectra which display the bands characteristic of these units, Fig. S1 The IR spectrum of SCP1 exhibits mainly medium and strong bands at 3064 and 3047 cm^-1^ (νCH), 1577, 1516 and 1480 cm^-1^ (νC=C), 1428 and 1333 cm^-1^ (δCH) and 732 and 695 cm^-1^ (γCH), attributed to the phenyl groups of the Ph_3_Sn connecting units which resemble the bands of Ph_3_SnCl. In addition to these phenyl characteristic bands, one should not ignore the absorption bands in 1958, 1882, 1818, 1768 and 1705 cm^-1^ which afford one of the best and most selective methods for the identification of benzene-ring substitution due to overtones. The presence of dmqox in the network structure of SCP1 is supported by the bands due to νCH, νC=N, νC=C, νC–N and γCH, Fig. S1. The bands due to νC=N and νC=C of dmqox exhibit shifts to lower wave numbers than those of dmqox itself supporting the coordination of dmqox ligand to the copper atoms. The Cu_2_(CN)_4_ fragment which represents the main building block of the structure of SCP1 absorbs at 2,113 and 2,085 cm^-1^ due to νC≡N. Also, it absorbs at 410 and 405 cm^-1^ due to νCu–C. interestingly, the presence of two νC≡N and two νCu–C bands in the IR spectrum of SCP1supports the presence of two types of the cyanide ligand in its structure. On the other hand, the νC≡N bands locate at wave numbers higher than the bands of genuine salts of the corresponding [Cu(CN)_4_]^-3^ anions indicating the presence of corrugated chains in the network structure of SCP1. In this case the network structure is constructed via Cu_2_(CN)_4_ building blocks connected by the Ph_3_Sn units creating corrugated chains of Sn–N:C–Cu–≡ CN–Sn. Thus, the structure of SCP1contains the Cu_2_(CN)_4_ fragments, the dmqox ligand as well as the Ph_3_Sn connecting units.


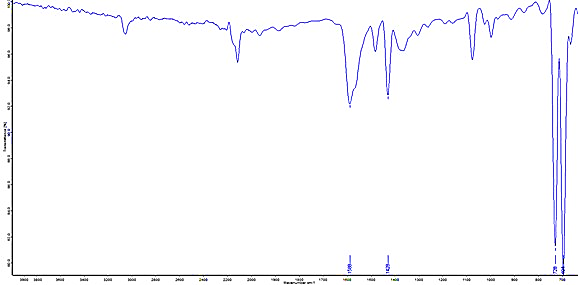


**Figure S1 IR Spectrum of SC1**

**2) ^1^H and ^13^C -NMR Spectra of ∞3[ Cu_2_(CN)_4_(Ph_3_Sn)_2_. dmqox]; “SCP1”**

The ^1^H-NMR spectrum of SCP1 shows one sharp singlet peak at 2.65 ppm which can be assigned to the six protons of the two methyl groups since they are chemically and magnetically equivalent, while the aromatic protons of the dmqox ligand give rise to two broad multiple peaks at 7.67–7.71 [H (6,7)] and 7.92–7.96 ppm [H (5,8)] as supported by the ratio of the integration of these peaks which equals to 6:3.91. This assignment was also supported by consulting the ^1^H-NMR spectrum of dmqox, which exhibits sharp singlet and two multiples at 2.63, 7.67–7.70 and 7.91–7.93 ppm corresponding to 2CH_3_, H (6,7) and H (5,8), respectively. The ^1^H-NMR spectrum of SCP1exhibits also, three broad peaks at 7.32, 7.43 and 7.78 ppm due to the absorption of the aromatic protons of the Ph_3_Sn units Fig. S2**.** These peaks are different in shape and position than those observed in the ^1^H-NMR spectrum of Ph_3_SnCl at 7.42–8.01 ppm. This observation indicates participation of the Ph_3_Sn units in the structure of SCP1as connecting units forming TBPY-5 configuration. Thus, ^1^H-NMR spectra of dmqox, Ph_3_SnCl and SCP1supported the presence of dmqox and the Ph_3_Sn in the structures of SCP1**.** The ^13^C-NMR spectrum of 1 displays sharp signals at 23.66, 128.89, 129.67, 141.16, 141.27, 154.77 and 154.89 ppm while that of 2 displays sharp signals at 23.66, 128.58, 128.89, 129.20, 129.67, 129.95, 136.92, 141.30, 143.07 and 154.89 ppm**,** Fig. S3. The ^13^C-NMR spectrum of SCP1 shows mainly the signals due to the resonance of dmqox, Ph_3_Sn connecting units and the cyanide ligands. The signals at 23.66 (Me), 128.89 (C6 and C7), 129.67 (C5 and C8), 141.27–141.30 (C9 and C10) and 154.89 (C2 and C3) ppm correspond to the resonance of the dmqox which appear at the same position as those of dmqox itself. On the other hand, the signals at 129.20, 129.95 and 136.92 ppm due to the Ph_3_Sn connecting fragments, in the spectrum of SCP1appear at the same position as those of the Ph_3_Sn fragment itself. The last sharp signal at 136.92 ppm exhibits two satellites at 136.47 and 137.37 ppm due to coupling with ^11SC2^n and ^11SC1^n nuclei. On the other hand, the cyanide ligand absorbs at 128.58 and 143.07 ppm indicating the presence of the cyanide ligand involved in the formation of the unique rhombic [Cu_2_(µ^3^- CN)_2_] motif and the cyanide ligand in the corrugated chain [CN–Ph_3_Sn–NC– Cu–CN], respectively. Thus, the ^13^C-NMR spectrum of SCP1 supports the fact that its network structure contains the rhombic [Cu2(µ^3^-CN)_2_] motif. The presence of the [Cu_2_(µ^3^-CN)_2_] fragments in SCP1 can be considered as an essential phenomenon in constructing such SCP1 which acquire large space to accommodate the organic voluminous ligands. The crystallographic data of SCP1 support this opinion where the reported expanded structure of **_∞_**^3^[Cu_2_(CN)_4_(Ph_3_Sn)_2_.dmqox] contains two non-equivalent cyanide ligands. Also, the NMR data indicate that dmqox ligands play the role of connecting the corrugated chains of [CN–Ph_3_Sn–NC–Cu–CN] via the Cu^1^ atoms.


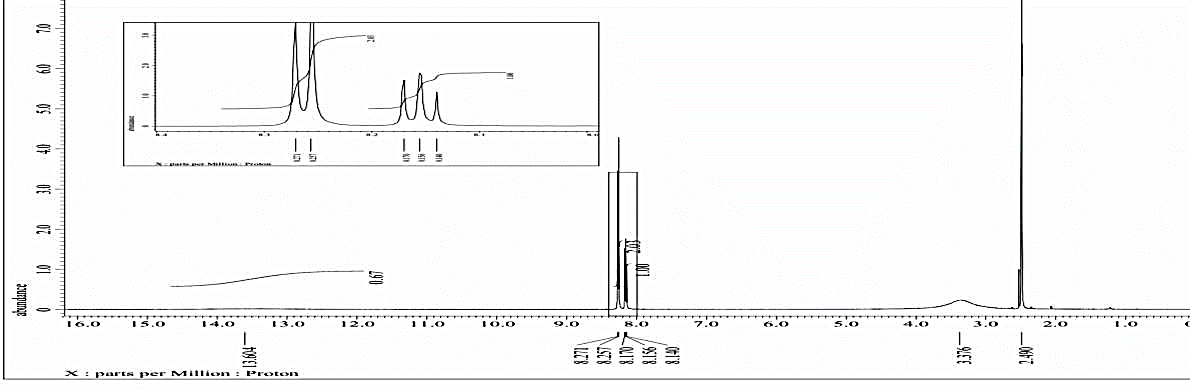


**Figure S2 The ^1^H-NMR Spectrum of SCP1.**


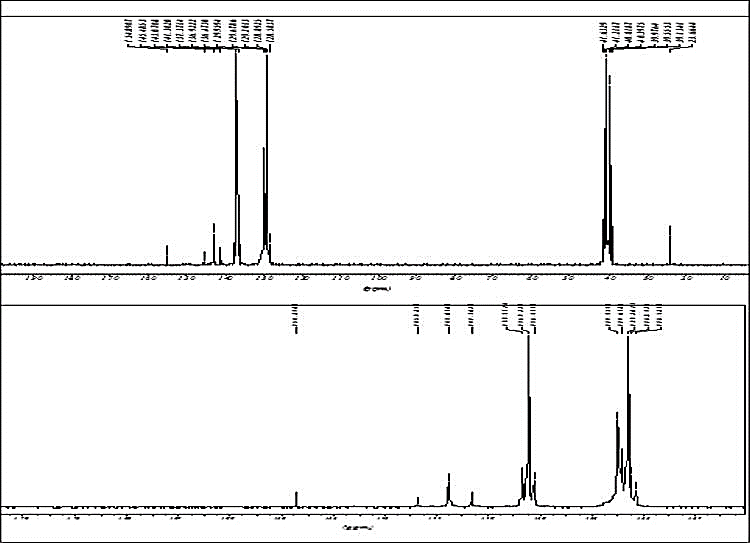


**Figure S3** ^13^C-NMR Spectrum of **SCP1**

**3) Mass Spectra of ^3^[ Cu_2_(CN)4(Ph_3_Sn)_2_. dmqox]; SCP1**

**∞**

The ESI^+^ mass spectrum of **SC1** exhibits the base peak at 351 corresponding to Ph_3_Sn^+^ isotopomer of the molecular ion. Fragmentation of dmqox ion gives rise to peaks at m/z = 50, 76, 102 and 117 due to [C_4_H_2_] ^+^, [C_6_H_4_]^+^, [C_7_H_4_N]^+^ and [C_8_H_7_N]+, respectively, Fig. S4. The mass spectrum of SCP1 shows the mass fragment corresponding to [Cu–dmqox]^+^ at m/z 222. The mass spectrum shows, also, the ion peaks at m/z =392, 503, 598, 717, 815 and 909 which can be attributed to the presence of [Cu(CN)_2_Ph_2_Sn]^+^,[Cu_2_CNPh_3_Sn]^+^, [CuCNPh_3_Sn(dmqox)]^+^,[Cu_2_(CN)_3_Ph_3_Sn(dmqox)]^+^,[Cu(CN)_2_(Ph_3_Sn)_2_]^+^and [Cu_2_(CN)_3_(Ph_3_Sn)_2_]^+^ fragments, respectively. In addition, the ion peaks due to [Cu_2_(CN)_4_Ph_3_Sn(dmqox) PhSn]^+^,[Cu(CN)2(Ph_3_Sn)_2_(dmqox)]^+^, [Cu_2_(CN)_3_Ph_3_Sn(dmqox) Ph_2_Sn]^+^, [Cu_2_(CN)_4_Ph_3_Sn(dmqox) Ph_2_Sn]^+^and [Cu_2_(CN)_2_(Ph_3_Sn)_2_(dmqox)]^+^ appear at m/z = 938, 973, 991, 1014 and 1034, respectively. Moreover, The peak of [Cu_2_(CN)_4_(Ph_3_Sn)_2_(dmqox)]^+^ ion at m/z 1086 is observed confirming the expected (M.W.) which can be considered as a further support of the chemical formula suggested by elemental analysis data. In addition, an ion peak at m/z1154 is observed due to Cu_2_(CN)_4_ (Ph_3_Sn)_2_PhSnCN] ^+^, Fig. S5. The ESI^-^ mass spectrum of SCP1 displays eleven peaks of [Cu_n_(CN)_n+1_]- units, where the base peak is observed at m/z 115 corresponding to [Cu(CN)_2_]- isotopomer of the molecular ion, Figure S4.Thus, the mass spectra of SCP1support the presence of Ph_3_Sn, CuCN and dmqox units in its structure as well as the polymeric nature of the [Cu_2_(CN)_4_] building blocks.


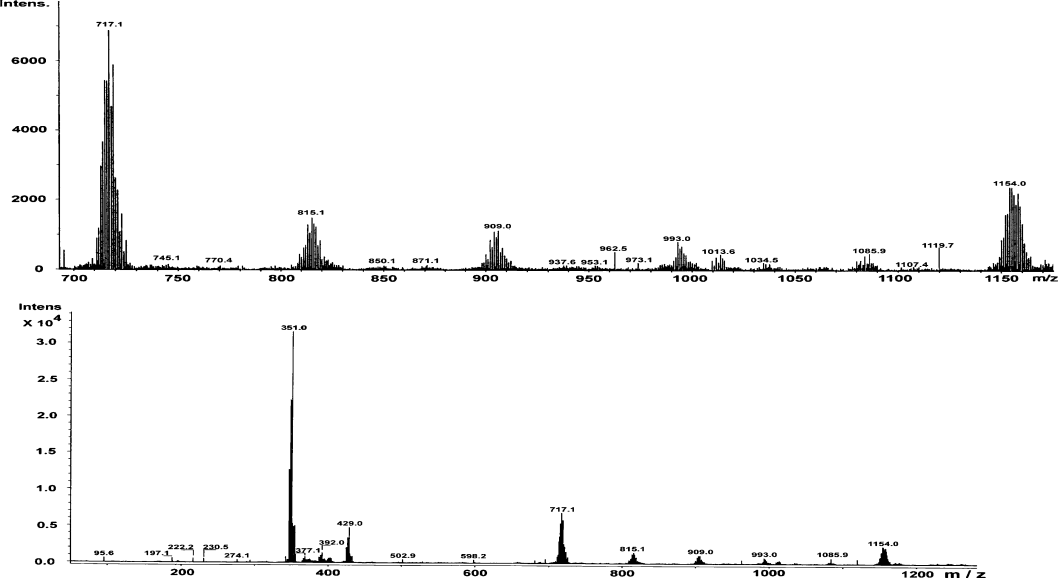


**Figure S4** Positive ion ESI mass spectrum of **SCP1**.


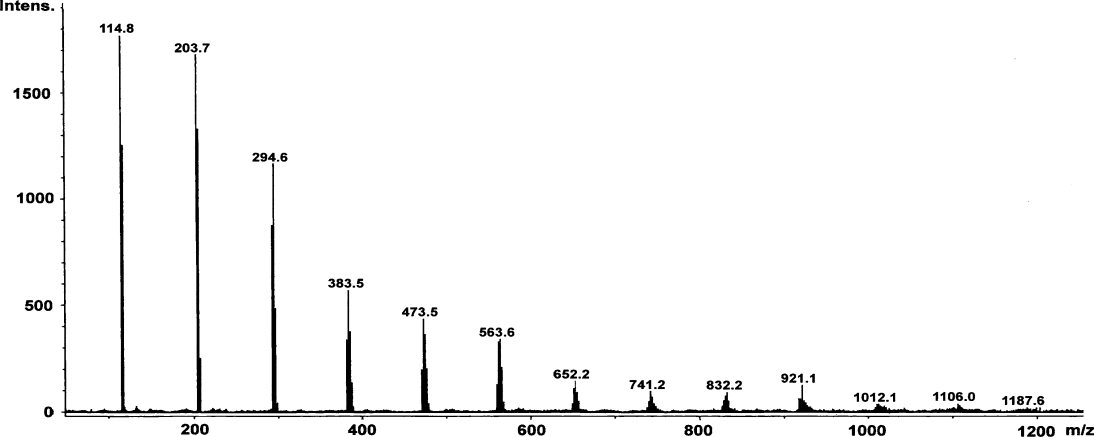


**Figure S5** Negative ion ESI mass spectrum of **SC1**. **X**-ray Powder Diffraction and Structure of SCP1

Spectroscopic data and elemental analyses show that SCP1has the chemical formula _∞_^3^[Cu_2_(CN)_4_(Ph_3_Sn)_2_. dmqox] which looks like that of ^3^_∞_[Cu2(CN)4(Ph3Sn)2qox], SCP1. The experimental X-ray powder and simulated diffract grams of SCP1and SCP2, respectively, have the same reflections, 2θ values and the lattice constants indicating that they are isostructural. Thus, the network structure of **SCP1** exhibits two different tetrahedral copper (1) sites forming two Cu (CN)_2_ building blocks bridged by the Ph_3_Sn units to create elongated corrugated chains. These chains are connected by the dmqox molecules creating 3D-network structure which contains four rhombic [Cu_2_(µ^3^-CN)_2_] motifs as gathered from the IR and ^13^C- NMR spectra of **SCP1**. The tin sites are coordinated to three phenyl groups and two cyanide ligands in axial positions via coordinate bonds forming TBPY-5 configuration. The network structure of the **SCP1** can be considered as consisting of repeating units of the [Cu_2_ (µ^3^-CN)_2_] motifs connected by the CN–Sn–NC spacer on one side and by the dmqox spacer on the other side forming 3D-network. In this case, three different kinds of rings are formed. The 3D-network structure of SCP1 acquires non-interpenetrating rings creating box-like structure with cavities suitable to accommodate the bulky phenyl groups. However, the structures of SCP1 and SCP2 may be compared with those of the formal congeners described as [Cu_n_ (CN)_m_(R_3_Sn) L]. It is evident that the structures of these SCPs exhibit great diversity depending on the shape and size of the ligand as well as the type of organizing fragment. Interestingly, the structures of SCP1 and SCP2 contain three fused different rings, the rhombic [Cu_2_(µ-CN)_2_], the 18-atomic [Cu_4_ (Ph_3_Sn)_2_(CN)_4_(L)_2_] and the 24-atomic [C_8_N_8_Cu_4_(Ph_3_Sn)_4_] rings, which create a non-interpenetrating 3D-network. These rings form a box-like structure with cavities suitable to accommodate the bulky phenyl groups. Two important features can be observed regarding these structures that the rigid (qox, dmqox) ligands create non-interpenetrating framework, which is favorable with the formation, in most cases, of box-like structure as well as the formation of the rhombic

[Cu_2_(µ-CN)_2_] motif.


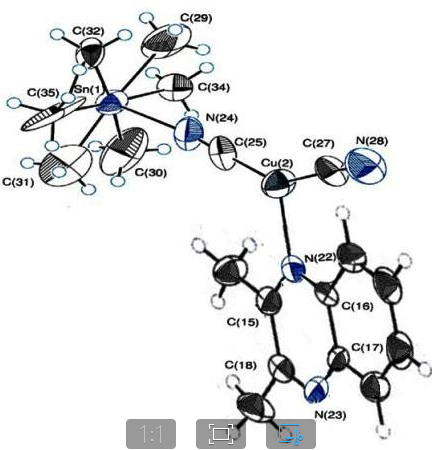


**Figure S6 Structure of _∞_^3^[ Cu_2_(CN)_4_(Ph_3_Sn)_2_. dmqox], SCP1. (Mercury 3.8 software was applied for Figure S6) (Mercury 3.8 software was applied for Figures S6)**

**Characterization and Structure of SCP2**

**4) Crystal Structure of ^3^∞[Cu_2_(CN)_4_·(CH_3_Sn)_2_·qaz], SCP2**

Self-assembly of the ternary adducts K_3_[Cu (CN)_4_], Me_3_SnCl and qaz in mixed solvents containing H_2_O and CH_3_CN at ambient conditions gives yellow prismatic crystals of the empirical composition ^3^∞[Cu(CN)_2_·Me_3_Sn·qaz] ≡ ^3^∞[Cu(CN)_2_·μ-Me_3_Sn μ-qaz], SCP2. The structure refinement parameters of SCP2 are tabulated in Table S1. The bond lengths and bond angles are collected in Table S2. The asymmetric unit of SC2 consists of one Cu^I^ atom, one trimethyl tin cation, one quinazoline (qaz) ligand and 2 CN ligands, Fig. S6 The Cu(I) atom locates at an inversion center and exhibits distorted trigonal plane (TP-3) topology by coordination to two ordered cyanide ligands and the N(22) atom of qaz. The angles of the TP-3 deviate than being 120° due to the close contacts between Cu(I) atom and C(26); Cu(2)–C(26) = 3.298 A˚ , the case which would adopt puckered zigzag chains containing the more acute angles. The ordered cyanide groups assume nearly linear configuration while the Cu–C distances are in the normal reported range, Table S2**.** The tin atom is coordinated to the methyl groups and two nitrogen atoms of two CN groups occupying axial positions coordinate to the Sn atom adopting triangle pyramid (TBPY-5) configuration. The methyl groups of the tin atoms turn out to have high thermal parameters due to rapid mobility of the methyl ligands. The Cu (CN)_2_ moiety can be considered the main building block of the structure of SCP2 where the trimethyl tin cation connects the building blocks creating 1D-infinite parallel zigzag chains. 2D-chains are constructed via interwoven of the 1D-chains which are stabilized by H-bonds (2.872-3.093 Ǻ), Fig. S8. The unprecedented bridging capability of the qaz ligand causes the structure to run three dimensionally creating the distorted 18-atom hexagonal ring structure, Fig.S7**,** where qaz ligand connects the chains via coordination of N22 to the Cu^I^ atom in one chain and through H- bonds utilizing the other N-atom and the methyl groups in another chain. The 3D-network is further stabilized by π–π stacking; 3.045–3.338 Ǻ Fig. S9.

**Table S1** Crystal data and structure refinement parameters of **SCP2,** ^3^ _∞_[Cu (CN)_2_·Me_3_Sn·qaz]

|  | **SCP2** |
| --- | --- |
| **Empirical Formula** | C13H15N4CuSn |
| **Formula Weight g/mol** | 409.527 |
| **Temperature (K)** | 298 |
| **Wavelenghth (Å)** | 0.71073 |
| **Crystal system** | Monoclinic |
| **Space group** | P21/c |
| **Unit cell dimensions** |  |
| **α (Å)** | 11.6055 (4) |
| **β (Å)** | 11.1671 (6) |
| **c(Å)** | 14.1182 (9) |
| **α\|( ** | 90.00 |
| **β( ** | 120 (18) |
| **γ( ** | 90.00 |
| **V(Å^3^ ** | 1596.45(15)/4 |
| ***D*calc. (g.cm^-3^ ** | 1.704 |
| **F(000)** | 692 |
| **ϴ-range (** | 2.910-27.485 |
| **Reflection collected/unique** | 11748/1138 |
| **Rint** | 0.062 |
| **Data/restraints/parameters** | 1138/0/194 |
| ***R* indices [*I* > 3 (*σ*)] *R_1_/wR_2_*** | 0.3070/0.146 |
| **W = 1/s^2^ (Fo^2^) + 0.10000 x Fo^2^)** |  |
| **R indices (all data)** | 0.253/0.184 |
| **Largest difference peak and hole (e Å^-3^)** | 2.67/-3.91 |

**Table S2** Selected bond lengths (A˚) and bond angles (°) of **SCP2**

| Sn(1)-N(24) | 2.289(6) | N(24)-Sn(1)-C(35) | 89.0(2) |
| --- | --- | --- | --- |
| Sn(1)-N(28) | 2.386(2) | N(24)-Sn(1)-N(28) | 179.30 |
| Sn(1)-C(30) | 2.13(2) | C(30)-Sn(1)-C(32) | 174.6(6) |
| Sn(1)-C(32) | 2.197(12) | C(30)-Sn(1)-C(29) | 116.8(8) |
| Sn(1)-C(29) | 2.18(2) | C(30)-Sn(1)-C(29) | 116.8(8) |
| Sn(1)-C(31) | 2.14(2) | C(30)-Sn(1)-C(35) | 122.5(6) |
| Sn(1)-C(34) | 2.302(12) | C(32)-Sn(1)-C(31) | 112.6(6) |
| Sn(1)-C(35) | 2.14(2) | C(32)-Sn(1)-C(34) | 124.1(5) |
| Cu(2)-N(22) | 2.066(6) | C(29)-Sn(1)-C(35) | 120.7(6) |
| Cu(2)-C(25) | 1.893(7) | C(31)-Sn(1)-C(34) | 123.3(5) |
| Cu(2)-C(27) | 1.868(6) | N(22)-Cu(2)-C(25) | 113.0(3) |
| C(27)-N(28) | 1.120(7) | N(22)-Cu(2)-C(27) | 107.4(3) |
| C(25)-N(24) | 1.158(7) | C(25)-Cu(2)-C(27) | 139.6(3) |
| Cu(2)-C(27)-N(28) | 175.1()7 | Cu(2)-N(22)-C(16) | 117.6(5) |
| N(24)-Sn(1)-C(30) | 90.9(4) | Cu(2)-N(22)-C(15) | 126.4(6) |
| N(24)-Sn(1)-C(32) | 92.8(4) | Cu(2)-C(25)-N(24) | 178.7(7) |
| N(24)-Sn(1)-C(29) | 86.7(5) | Sn(1)-N(24)-C(25) | 171.9(6) |
| N(24)-Sn(1)-C(31) | 87.3(4) | Sn(1)-N(28)-C(27) | 173.07 |
| N(24)-Sn(1)-C(34) | 91.4(3) | - | - |


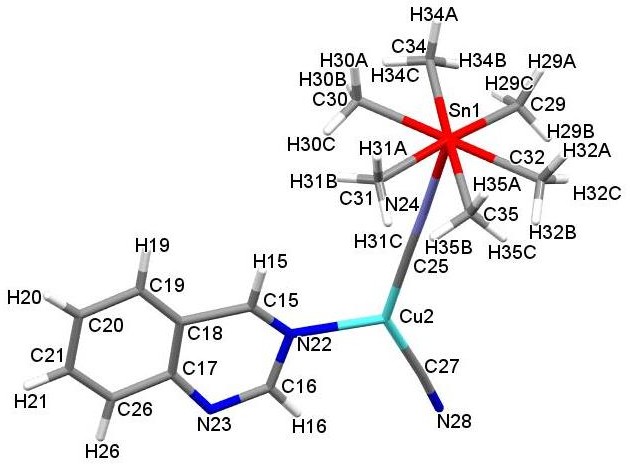


**Figure S7** Perspective view of the asymmetric unit of the **SCP2**, showing the atom labeling scheme (Mercury 3.8 software was applied for Figures S7)


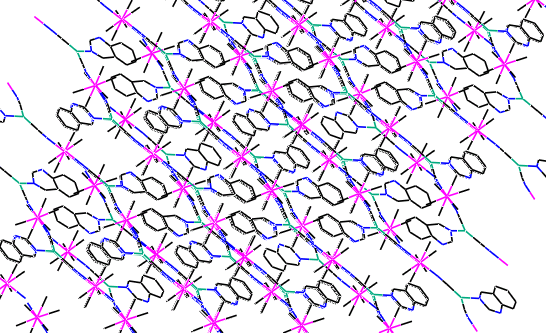


**Figure S8** A view of the interwoven 2D-sheets of the **SCP2** in the ab-plane developing 3D-network structure. H atoms are omitted for clarity.


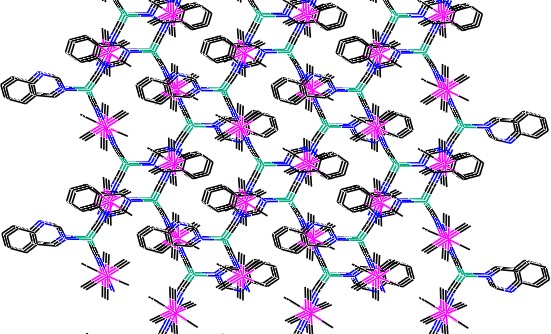


**Figure S9** Visualization of the 3D-network structure of the **SCP2** down the projection of b-axis showing the hexagonal ring structure.

References

1. S. E. H. Etaiw, S. N. Abdou; Journal of Inorganic and Organometallic Polymers and Materials, 22(4) (2012) 780.
2. S. E. H Etaiw, E. Mohamed, T.A. Fayed, S. N. Abdou; Journal of Inorganic and Organometallic Polymers and Materials, 21(3) (2011) 480.
3. L. Ronconi, C. Marazano, U. Russo, S. Sitran, R. Coraziani, D. Fregona; Appl. Organomet. Chem., 17 (2003) 9.
4. H. Hanika-Heidl, S. E. H. Etaiw, M. S. Ibrahim, A.S. Badr El-din, R.D. Fischer; J. Organomet. Chem., 684 (2003) 329.
5. S. E. H. Etaiw, S. N. Abdou; J. Inorg. Organomet. Polym. 20 (2010) 622.
6. S.E.H. Etaiw, S.N. Abdou; J. Inorg. Organomet. Polym. 20 (2010) 622.
7. R. J. Williams, A. C. Larson and D. T. Cromer; Acta Cryst. B,28 (1972)858.
8. S.E.H. Etaiw, S.N. Abdou, A. S. Badr El-din; J. Inorg. Organomet. Polym. 25, (2015)1394.
